# Cytoneme-like protrusion formation induced by LAR is promoted by receptor dimerization

**DOI:** 10.1242/bio.059024

**Published:** 2022-07-25

**Authors:** Mai Quynh Nguyen, Manabu Taniguchi, Misato Yasumura, Tokuichi Iguchi, Makoto Sato

**Affiliations:** 1 Graduate School of Frontier Biosciences, Osaka University, Osaka 565-0871, Japan; 2 Department of Anatomy and Neuroscience, Graduate School of Medicine, Osaka University, Osaka 565-0871, Japan; 3 Department of Nursing, Faculty of Health Science, Fukui Health Science University, Fukui 910-3190, Japan; 4 Division of Developmental Neuroscience, Department of Child Development, United Graduate School of Child Development, Osaka University, Kanazawa University, Hamamatsu University School of Medicine, Chiba University and University of Fukui (UGSCD), Osaka University, Osaka 565-0871, Japan

**Keywords:** Leukocyte common antigen-related receptor, Tunneling nanotube, RPTP, Receptor protein tyrosine phosphatase

## Abstract

Actin-based protrusions called cytonemes are reported to function in cell communication by supporting events such as morphogen gradient establishment and pattern formation. Despite the crucial roles of cytonemes in cell signaling, the molecular mechanism for cytoneme establishment remains elusive. In this study, we showed that the leukocyte common antigen-related (LAR) receptor protein tyrosine phosphatase plays an important role in cytoneme-like protrusion formation. Overexpression of LAR in HEK293T cells induced the formation of actin-based protrusions, some of which exceeded 200 µm in length and displayed a complex morphology with branches. Upon focusing on the regulation of LAR dimerization or clustering and the resulting regulatory effects on LAR phosphatase activity, we found that longer and more branched protrusions were formed when LAR dimerization was artificially induced and when heparan sulfate was applied. Interestingly, although the truncated form of LAR lacking phosphatase-related domains promoted protrusion formation, the phosphatase-inactive forms did not show clear changes, suggesting that LAR dimerization triggers the formation of cytoneme-like protrusions in a phosphatase-independent manner. Our results thus emphasize the importance of LAR and its dimerization in cell signaling.

This article has an associated First Person interview with the first author of the paper.

## Abstract (precis)

**Summary:** We showed that the formation of cytoneme-like protrusions, which function in cell signaling, is induced by LAR and clarified that it is LAR dimerization which promotes protrusion formation.

## INTRODUCTION

In addition to the juxtacrine signaling type of contact-dependent cell-to-cell communication, the formation of thin membrane protrusions that connect cells located far apart from each other warrants attention. Currently, these thin cellular protrusions are structurally classified into three major groups based on their cytoskeletal components and on their tip shapes (Yamashita et al., 2018). The first group encompasses open-ended and actin-based connections termed tunneling nanotubes (TNTs) that enable the exchange of soluble cytoplasmic components and intracellular vesicles between connecting cells (Gerdes and Carvalho, 2008; Rustom, 2016; Rustom et al., 2004). The other two groups encompass closed-ended microtubule-based protrusions called MT nanotubes (Inaba et al., 2015) and closed-ended actin-based protrusions called signaling filopodia or cytonemes (González-Méndez et al., 2019).

Actin-based thin specialized signaling filopodia, which are receiving increasing attention regarding their contributions to various cellular events, were first described in a study of sea urchin gastrulation (González-Méndez et al., 2019; Miller et al., 1995) and were termed cytonemes (‘cytoneme’ means thread-like cytoplasm) in a study on *Drosophila* imaginal discs (Ramírez-Weber and Kornberg, 1999). To date, in addition to the different tissue types in *Drosophila*, cytonemes have been observed in zebrafish embryo neural plates (Mattes et al., 2018; Stanganello et al., 2015), blastulas (Caneparo et al., 2011) and epidermal tissues (Hamada et al., 2014); in chick embryo somites (Sagar et al., 2015) and limb buds (Sanders et al., 2013); and in mouse embryo blastocysts (Salas-Vidal and Lomelí, 2004). Interestingly, cytoneme-like protrusion induction has also been reported to occur in HEK cells with the expression of the stem cell markers Lgr5 and Lgr4 (Snyder et al., 2015). These findings reveal the common characteristics of cytonemes: they are rich in actin, with some also containing microtubules; have lengths ranging from 0.25 µm to up to 380 µm; and have varying extension and retraction dynamicity (González-Méndez et al., 2019; Mattes and Scholpp, 2018). Previous studies have also shed light on the roles of cytonemes in cell signaling, including establishment of morphogen gradients (hedgehog and Wnt delivery) (Bischoff et al., 2013; Mattes et al., 2018; Stanganello et al., 2015), pattern formation (spatial inhibition of Notch signaling to produce an ordered bristle pattern) (Cohen et al., 2010; De Joussineau et al., 2003), directed morphogenesis (Huang and Kornberg, 2015), and maintenance of the stem cell niche (Fuwa et al., 2015; Mandal et al., 2007; Rojas-Ríos et al., 2012). Regarding cytoneme formation, in the most studied tissue, the *Drosophila* wing disc, filopodial polarization is suggested to be controlled by the formation of the Rho GTPase Rac gradient (Couto et al., 2017; Georgiou and Baum, 2010). Vesicle sorting is also proposed to play an important role in the basolateral formation of cytonemes by transporting signaling ligands to the basolateral side (Bilioni et al., 2013; Callejo et al., 2011; Sohr et al., 2019). In zebrafish, local cytoneme nucleation is induced by the Wnt8a-dependent recruitment of CDC42-dependent assembly protein 1 (Ho et al., 2004; Stanganello et al., 2015). However, a complete understanding of the molecular mechanism for cytoneme establishment and functional maintenance is lacking, especially in mammals.

In this study, we investigated whether the leukocyte common antigen-related (LAR) receptor protein tyrosine phosphatase, which appears to be crucial for the growth of axons – one type of protrusion for cell signaling – and for nerve regeneration (Fisher et al., 2011; Johnson et al., 2001; Xie et al., 2001; Xu et al., 2015), has roles in the formation of cytonemes or other types of thin protrusions. LAR belongs to the type IIa RPTP subfamily of the receptor protein tyrosine phosphatase (RPTP) superfamily. The type IIa RPTP subfamily consists of three members in vertebrates (LAR, PTPσ, PTPδ), two in *Drosophila* (DLAR and RPTP69D), and one in *C. elegans* (PTP-3) (Ackley et al., 2005; Coles et al., 2015). All of these members possess a common organization of structural domains comprising extracellular adhesion-like domains [three Ig-like domains and a modifiable number of fibronectin type III (FN1-9) repeats], a transmembrane region, and two tandem intracellular phosphatase domains (Coles et al., 2015; Stoker, 1994). They also have multiple variants generated through alternative splicing. Splicing extracellular FN4, FN5, FN6, and FN7 together with four mini-exon (meA, meB, meC, and meD) inserts creates various type IIa RPTP isoforms (Pulido et al., 1995), and the expression of these variants is tissue-specific throughout the course of development (Pulido et al., 1995; Wang et al., 1995). As reflected in their names, the type IIa RPTPs were initially focused on their phosphatase enzymatic activity. The reported LAR substrates include insulin receptor (Hashimoto et al., 1992), epidermal growth factor receptor (Hashimoto et al., 1992), beta-catenin (Müller et al., 1999), and EphA2 (Lee and Bennett, 2013) that are involved in cell migration. Later, however, the interaction of the type IIa RPTPs with various ligands in synapse formation and their dimerization or clustering control in molecular signaling received increasing attention. Presynaptic LAR is reported to interacts with postsynaptic netrin-G ligand-3 (NGL-3) (Kwon et al., 2010) in shaping synapse development. The extracellular matrices compositions, such as heparan sulfate proteoglycans (HSPGs) and chondroitin sulfate proteoglycans (CSPGs), are reported to interact with RPTP family controlling intermolecular clustering of these receptors and affecting neurite growth (Coles et al., 2011).

Complementing other studies, this study reveals that LAR has the potential to induce cytoneme-like protrusion formation in mammalian cells and that its dimerization or clustering – controlled by heparan sulfate – is crucial for long and branching protrusion formation. These findings suggest the possible existence of a common molecular mechanism for membrane protrusion formation during cell-to-cell communication establishment, a process in which LAR is an indispensable key player.

## RESULTS

### LAR overexpression in mammalian cells induced protrusion formation

**Fig. 1.**
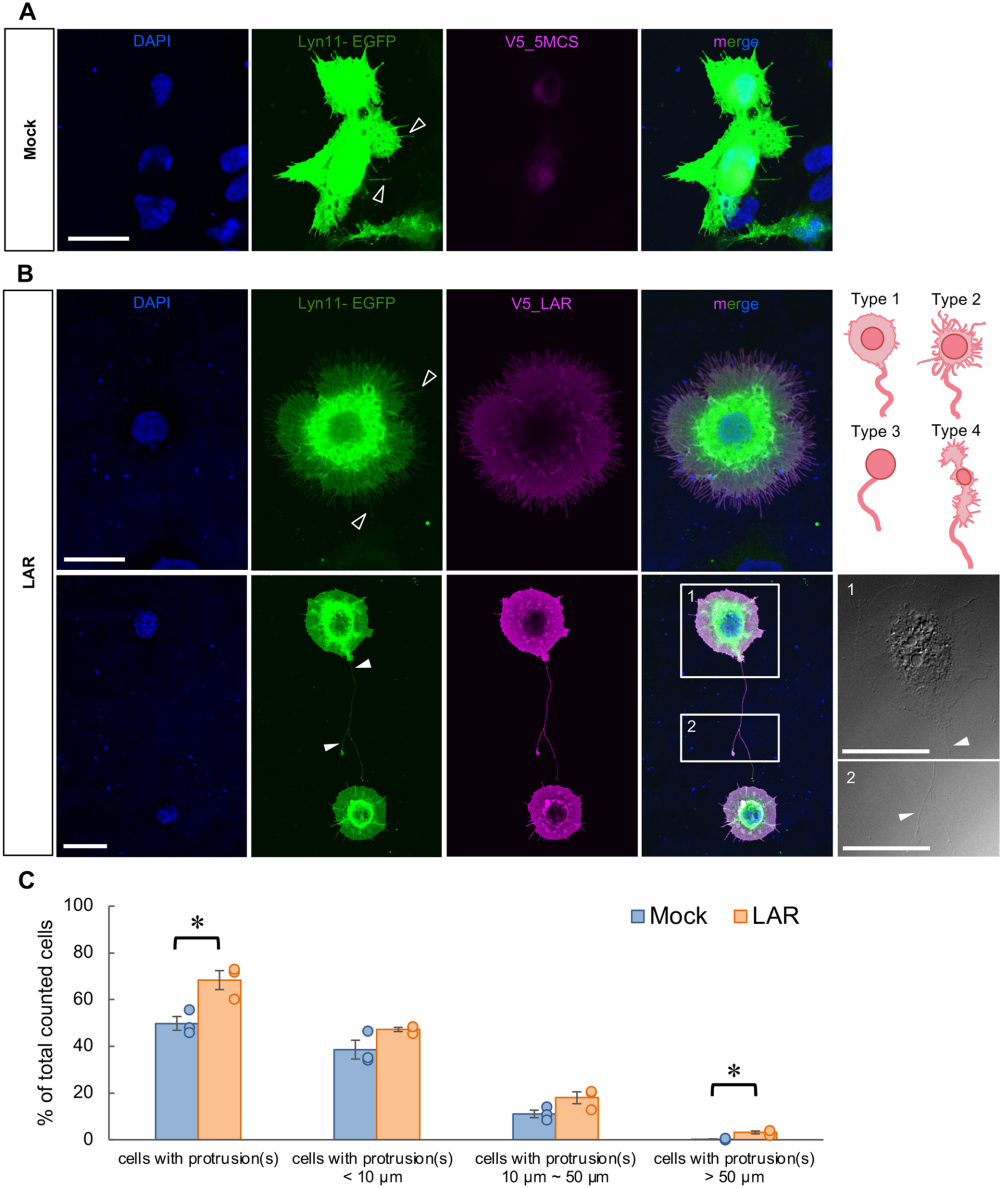
**LAR induced protrusion formation in mammalian cells.** (A) Representative cells from three independent trials of control (mock) vector expression in HEK293T cells. DAPI: blue, Lyn11-EGFP: green, V5: magenta. Open arrowheads: short protrusions. Scale bar: 20 µm. (B) Representative cells from three independent trials of LAR overexpression in HEK293T cells. Upper row: cell with multiple short protrusions. Lower row: cell with long and branching protrusion. Insets 1 and 2 are DIC images of the cell soma and the branching point and tips of the long protrusion on the cell. The major types of soma-shape of the cells with dominantly long protrusion are depicted on the right most of the upper row. DAPI: blue, Lyn11-EGFP: green, V5: magenta. Open arrowheads: short protrusions, arrowheads: long protrusion. Scale bars: 20 µm. (C) Cell-count analysis of the percentage of cells with protrusions in mock vector- and LAR-expressing samples. *N*=3 independent trials; total counted cell number *n*=457 and 819 cells for the mock and LAR samples, respectively. *P<0.05, Student's *t*-test. Mean±s.e.m. The dots denote the average value calculated from each trial.

LAR overexpression was found to induce the formation of protrusions in the HEK293T cell line, which was previously reported to form cytoneme-like structures by the overexpression of Lgr4 and Lgr5 stem cell markers (Snyder et al., 2015). Compared to transfection of the control vector (mock vector) (Fig. 1A), transfection of the LAR-expressing vector (Fig. 1B) into HEK293T cells promoted the formation of protrusions. LAR-expressing cells were found to possess multiple short protrusions (Fig. 1B, upper row), and there are cells with dominantly long and branching protrusions (Fig. 1B, lower row). The soma shape of the LAR-expressing cells with dominantly long protrusion can be categorized into four major types: type 1 with cytoplasm expanding evenly surrounding the nucleus resulting in a round shape soma, type 2 with ‘filamentous’ cytoplasm surrounding the nucleus, type 3 with reduced cytoplasm volume covering only the nucleus, and type 4 with the cytoplasm expanding to both sides or to a single side of the nucleus (Fig. 1B). The percentage of LAR vector-transfected cells forming protrusion(s) (68.3%) was significantly higher than that of mock vector-transfected cells (49.8%) (Fig. 1C). In addition, while very few mock vector-transfected cells had protrusion(s) longer than 50 µm (0.2%), a significantly greater number of LAR vector-transfected cells had protrusion(s) longer than 50 µm (3.2%) (Fig. 1C).

### LAR-induced protrusions contained actin as a major cytoskeletal component

**Fig. 2.**
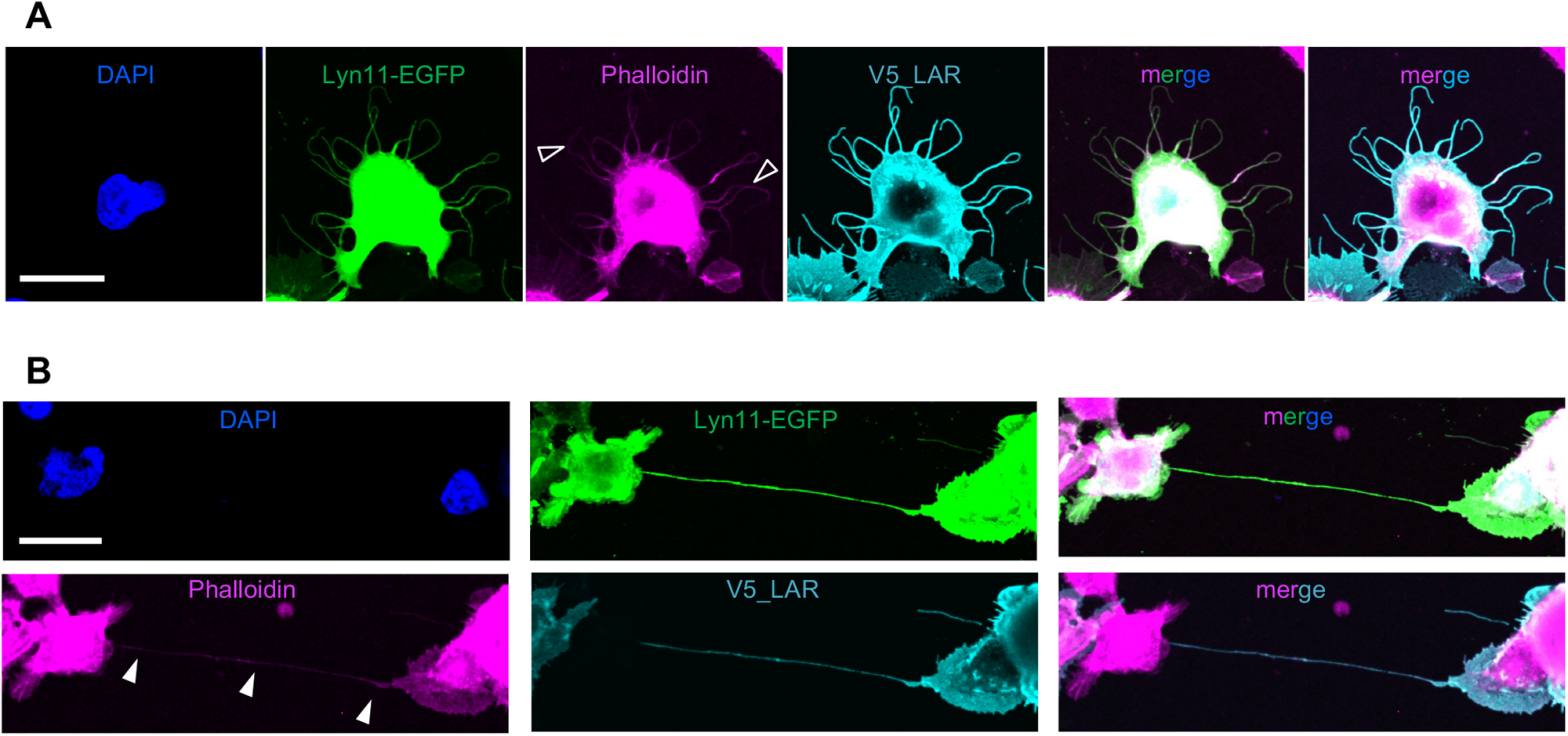
**LAR-induced protrusions contained actin.** (A) Representative images of actin staining from three independent trials showing a cell with short protrusions. DAPI: blue, Lyn11-EGFP: green, phalloidin: magenta, V5_LAR: cyan. The open arrowheads point to the short protrusions. Scale bar: 20 µm. (B) Representative images of actin staining from three independent trials showing a cell with dominantly long protrusion. DAPI: blue, Lyn11-EGFP: green, phalloidin: magenta, V5_LAR: cyan. The arrowheads point to the formed long protrusion. Scale bar: 20 µm.

To investigate the cytoskeletal component of LAR-induced protrusions, we examined whether these protrusions contained actin (by immunocytochemical staining using fluorescently tagged phalloidin) and microtubule (by immunocytochemical staining against alpha tubulin) or not. The results showed that the formed protrusions induced by LAR overexpression contained actin. Fluorescence signals were detected in both the short protrusions (less than 50 µm in length) (Fig. 2A) and the long protrusions (more than 50 µm in length) (Fig. 2B). The immunocytochemical staining of alpha tubulin, however, showed clear signals of tube-like structures within the cell soma, but very weak dot-like signals within the formed protrusions (Fig. S1). Electron microscopic images of a LAR and Lyn11-EGFP co-transfected cell also showed presence of microtubule at the base, and presence of actin fibers within the formed protrusion (Fig. S2). Further, the electron microscopic images of a LAR_ΔD1D2 (the truncated form of LAR lacking D1 and D2 domains) and Lyn11-EGFP co-transfected cell, revealed presence of macrovesicle in addition to actin fiber within the observed long protrusion, but no presence of microtubule (Fig. S3).

In addition to the immunocytochemical and electron microscopic results, time-lapse imaging of LAR and Lyn11-EGFP co-transfected cells treated with cytochalasin D, an actin polymerization inhibitor, also confirmed the importance of an actin cytoskeletal composition in the observed protrusions. Dynamicity, evaluated as the growth and/or shrinking speed, was significantly higher in protrusions formed in LAR and Lyn11-EGFP co-transfected cells treated with vehicle (DMSO) (Movie 1) and non-treated cells (average of 0.79 µm min^−1^ and 0.74 µm min^−1^, respectively) than in cells treated with cytochalasin D (average of 0.21 µm min^−1^) (Movie 3), indicating that blockade of actin polymerization affected protrusion formation (Fig. S4). This inhibitory effect of cytochalasin D on the observed protrusion dynamicity was attenuated after washout in one cell (Movie 4) out of the total of eight cells (the other seven cells showed quick retraction and/or segmentation of the observed protrusions after washout, probably due to the medium renewal, which is known to cause cell detachment even in the case of healthy intact cells). Treatment of nocodazole, a microtubules-polymerization interferer, did not result into significant change in dynamicity of the observed protrusions (average of 0.50 µm min^−1^) (Movie 2) (Fig. S4). It is important to note that differ to the cytochalasin D (2 μM) application where cell survival was not apparently reduced after treatment, bath application of the same concentration of nocodazole (2 μM) caused up to about 50-70% cells detachment from the culture surface at 8 h post treatment.

### Rapamycin-induced LAR dimerization promoted protrusion formation

**Fig. 3.**
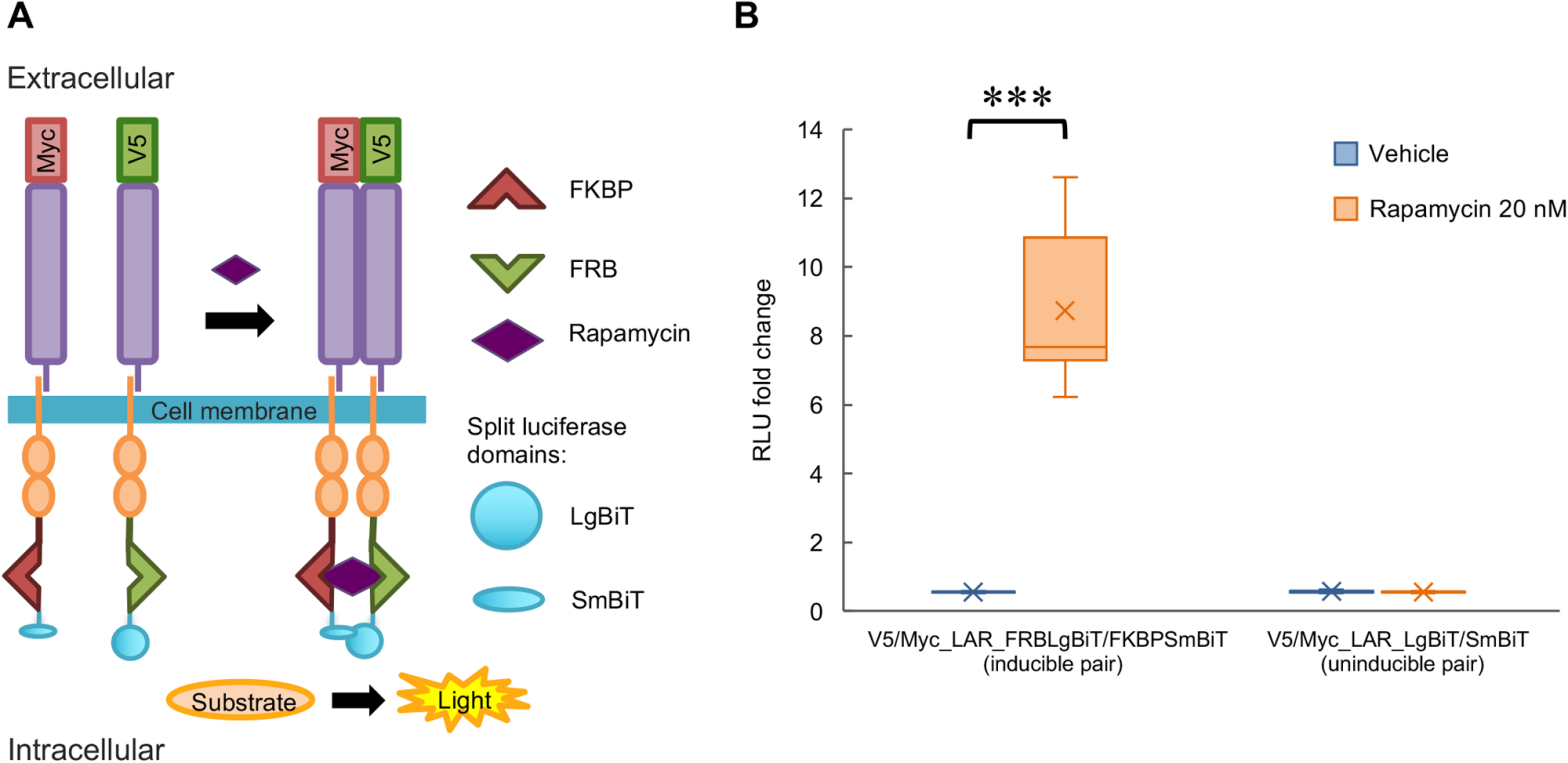
**Established rapamycin-induced LAR dimerization system.** (A) Schematic illustration of the rapamycin-induced LAR dimerization system. In this system, FRB and the larger domain of the split luciferase protein (LgBiT) were fused to the C-terminus of V5-tagged LAR, while FKBP and the smaller domain of the split luciferase protein (SmBiT) were fused to the C-terminus of Myc-tagged LAR. Interaction of rapamycin with FRB and FKBP induces LAR dimerization – a process that can be detected according to the reconstitution and functional restoration of the luciferase enzyme in the presence of its substrate. FKBP: FK506-binding protein, FRB: FKBP–rapamycin binding domain of mammalian target of rapamycin (mTOR). (B) Analytical results of the changes in detected luminescence upon rapamycin application to induce LAR dimerization followed by reconstitution and restoration of luciferase enzymatic activity (at 60 min post application). Inducible pair: V5_LAR_FRBLgBiT+Myc_LAR_FKBPSmBiT. Uninducible pair: V5_LAR_LgBiT+Myc_LAR_SmBiT. *N*=3 independent trials; total *n*=18 replicates for each sample type. ***P<0.0001, nonparametric Wilcoxon test. In the box plot, the horizontal line within each box denotes the median value, and the x mark denotes the mean value. RLU: relative light unit.

To investigate the importance of LAR dimerization or clustering for receptor function and its involvement in cytoneme-like protrusion formation, we established a system for LAR dimerization induction using rapamycin interactions with the FK506 binding protein (FKBP) and the FKBP–rapamycin binding (FRB) domain of mammalian target of rapamycin (mTOR) in combination with a split luciferase assay for dimerization detection. In this system, FRB or FKBP was fused to the C-terminus of LAR (tagged with V5 or Myc in the N-terminus) followed by LgBiT or SmBiT (the split domains of luciferase) so that when these constructs were expressed in HEK293T cells, the instantaneous formation of the FKBP-rapamycin-FRB complex upon rapamycin application would induce physical dimerization of the fused LAR proteins. Simultaneously, the split luciferase domains would be brought into close contact for reconstitution and activation of the luciferase enzymatic ability, which would be detectable with substrate application (Fig. 3A). Taking into account the fact that LARs themselves have the ability to dimerize independent of rapamycin, this basal dimerization status was confirmed prior to rapamycin application. Then, the successful induction of LAR dimerization was evaluated based on the difference in luciferase activity before versus after rapamycin application. The results clearly showed a significant increase in the detected luminescence amount post rapamycin (20 nM) application to the cells expressing the inducible pair (the pair of LARs fused with FRBLgBiT and FKBPSmBiT) but not in the cells expressing the uninducible pair (the pair of LARs with only LgBiT and SmBiT fusion) (Fig. 3B).

**Fig. 4.**
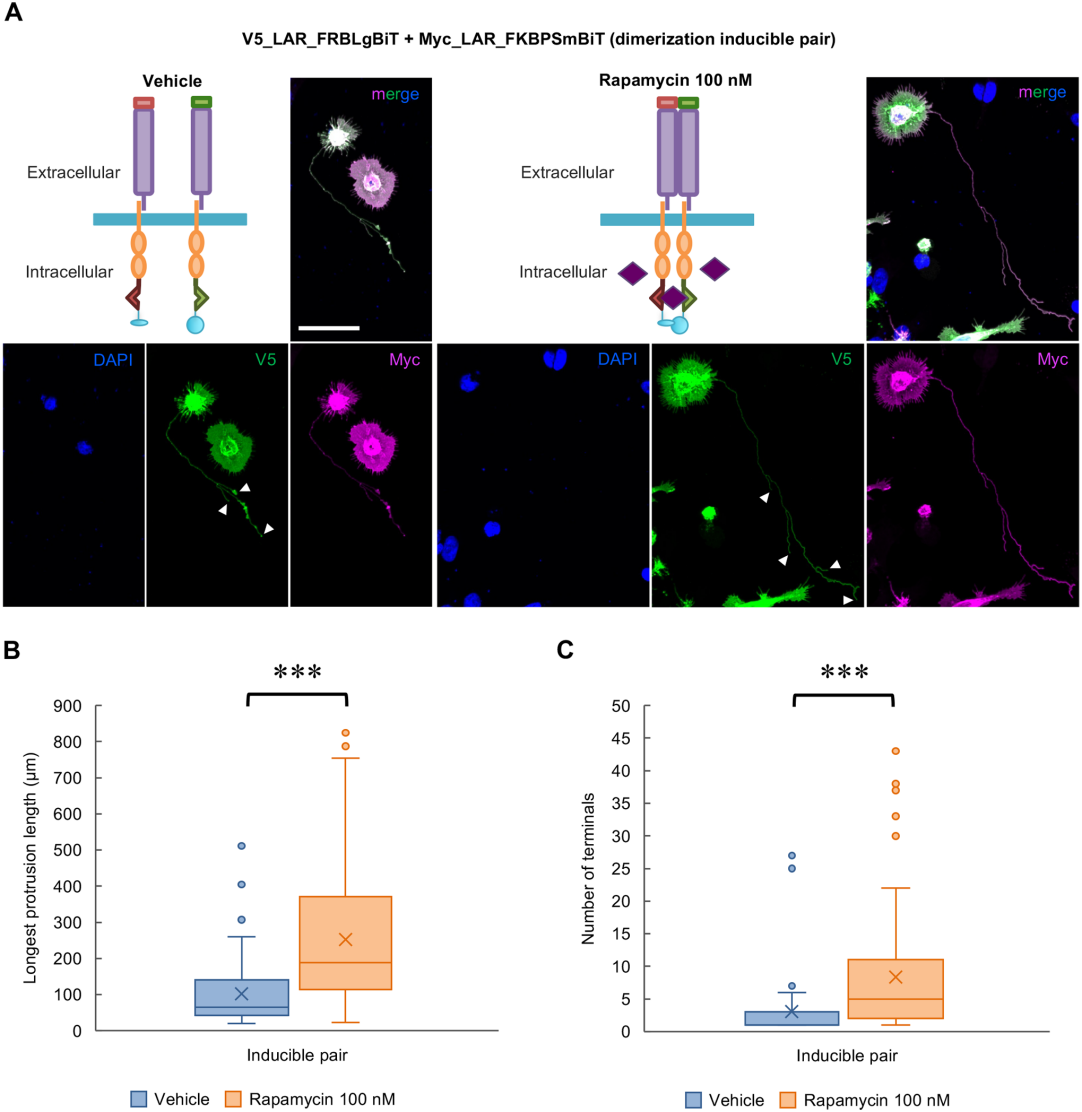
**Rapamycin-induced LAR dimerization promoted protrusion formation.** (A) Images of cells expressing the dimerization system inducible pair of constructs that were treated with vehicle or rapamycin (100 nM). DAPI: blue, V5: green, Myc: magenta. Arrowheads: counted protrusion end tips (terminals). Scale bar: 40 µm. (B) Analytical results of the longest protrusion length. *N*=3 independent trials; total *n*=58 and 87 cells, respectively. ***P<0.0001, nonparametric Wilcoxon test. In the box plot, the horizontal line within each box denotes the median value, and the x mark denotes the mean value. (C) Analytical results of the longest protrusion complexity. *N*=3 independent trials; total *n*=58 and 87 cells, respectively. ***P<0.0001, nonparametric Wilcoxon test. In the box plot, the horizontal line within each box denotes the median value, and the x mark denotes the mean value.

Following the confirmation of the successful induction of LAR dimerization with rapamycin in HEK293T cells via luciferase assay, we investigated whether the induced LAR dimerization promotes protrusion formation. We found that with rapamycin (100 nM) application, the cells expressing the inducible pair of LARs exhibited significantly longer (Fig. 4A,B) and more branched (Fig. 4A,C) protrusions than the vehicle-treated cells. This effect of rapamycin was not observed when rapamycin was applied to cells expressing the uninducible pair of LARs or to cells that expressed only one component of the inducible pair (V5_LAR_FRBLgBiT) (Fig. S5A,B,C). In the absence of LAR expression, treatment of rapamycin (100 nM) to the Lyn11-EGFP-expressing cells also did not show any changes (Fig. S5D). These results showed that rapamycin-induced dimerization of LAR FRBLgBiT/FKBPSmBiT fusion proteins was important for protrusion formation.

### LAR-induced protrusion formation was promoted in the presence of heparan sulfate

We next examined the regulation of LAR clustering, which is proposed to be the mechanism for the control of type IIa RPTPs in neurite outgrowth promotion, with the naturally existing extracellular matrix components heparan sulfate (HS) and chondroitin sulfate (CS).

**Fig. 5.**
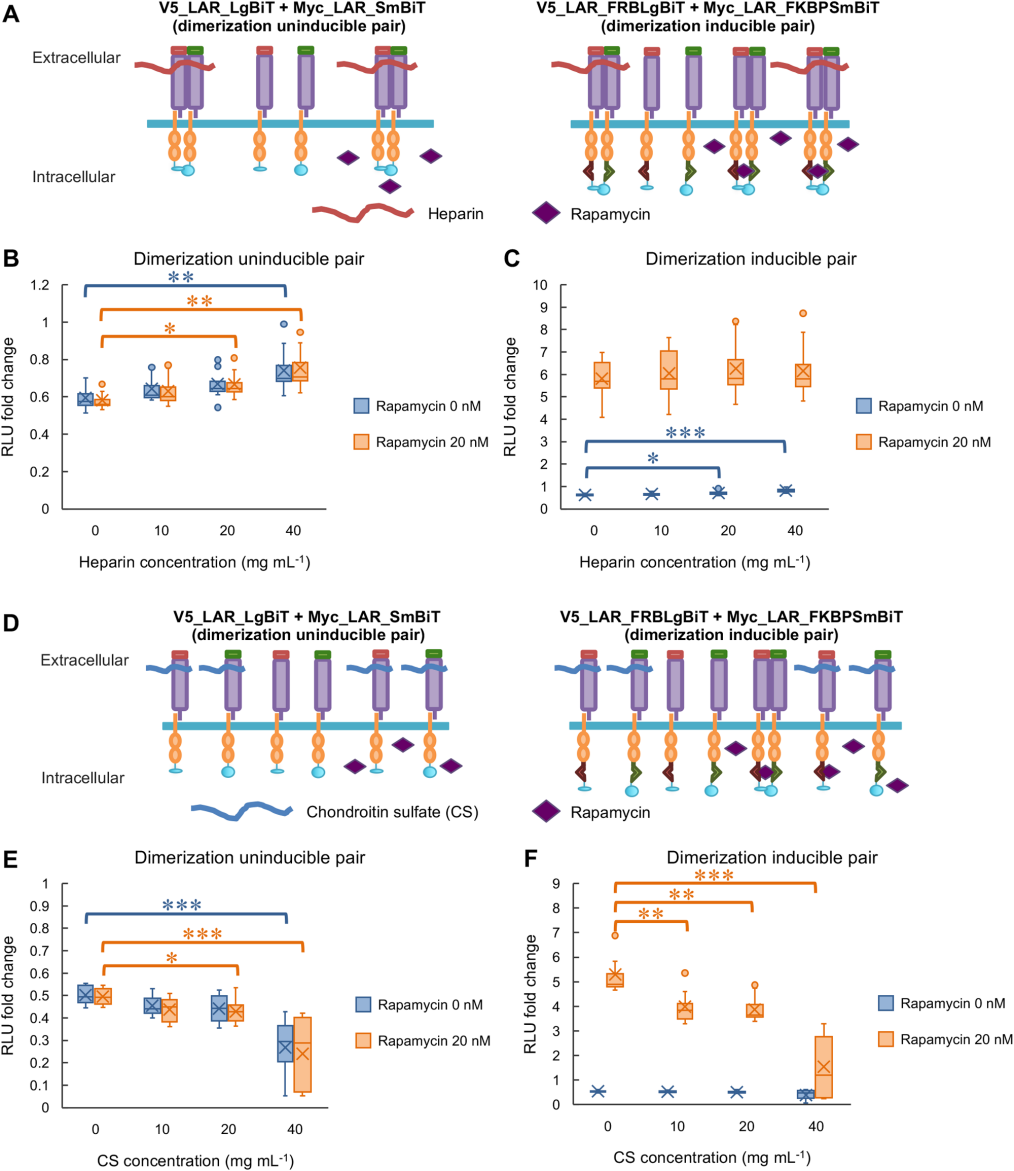
**Heparin promoted while chondroitin sulfate inhibited LAR dimerization.** (A) Schematic illustration of the effects of heparin and/or rapamycin application to dimerization uninducible pair (V5_LAR_LgBiT+Myc_LAR_SmBiT) or to dimerization inducible pair (V5_LAR_FRBLgBiT+Myc_LAR_FKBPSmBiT). (B) Analytical results of the changes in detected luminescence upon heparin and/or rapamycin application to induce LAR dimerization followed by reconstitution and restoration of luciferase enzymatic activity (at 60 min post application). Uninducible pair: V5_LAR_LgBiT+Myc_LAR_SmBiT. *N*=4 independent trials; total *n*=12 replicates for each sample type. *P<0.05 and **P<0.01, nonparametric Steel test. In the box plot, the horizontal line within each box denotes the median value, and the x mark denotes the mean value. RLU: relative light unit. (C) Analytical results of the changes in detected luminescence upon heparin and/or rapamycin application to induce LAR dimerization followed by reconstitution and restoration of luciferase enzymatic activity (at 60 min post application). Inducible pair: V5_LAR_FRBLgBiT+Myc_LAR_FKBPSmBiT. *N*=4 independent trials; total *n*=12 replicates for each sample type. *P<0.05 and ***P<0.001, nonparametric Steel test. In the box plot, the horizontal line within each box denotes the median value, and the x mark denotes the mean value. RLU: relative light unit. (D) Schematic illustration of the effects of chondroitin sulfate and/or rapamycin application to dimerization uninducible pair (V5_LAR_LgBiT+Myc_LAR_SmBiT) or to dimerization inducible pair (V5_LAR_FRBLgBiT+Myc_LAR_FKBPSmBiT). (E) Analytical results of the changes in detected luminescence upon chondroitin sulfate and/or rapamycin application to induce LAR dimerization followed by reconstitution and restoration of luciferase enzymatic activity (at 60 min post application). Uninducible pair: V5_LAR_LgBiT+Myc_LAR_SmBiT. *N*=4 independent trials; total *n*=12 replicates for each sample type. *P<0.05 and ***P<0.001, nonparametric Steel test. In the box plot, the horizontal line within each box denotes the median value, and the x mark denotes the mean value. RLU: relative light unit. (F) Analytical results of the changes in detected luminescence upon chondroitin sulfate and/or rapamycin application to induce LAR dimerization followed by reconstitution and restoration of luciferase enzymatic activity (at 60 min post application). Inducible pair: V5_LAR_FRBLgBiT+Myc_LAR_FKBPSmBiT. *N*=4 independent trials; total *n*=12 replicates for each sample type. **P<0.01 and ***P<0.001, nonparametric Steel test. In the box plot, the horizontal line within each box denotes the median value, and the x mark denotes the mean value. RLU: relative light unit.

Using the established rapamycin-induced LAR dimerization detection system, we reconfirmed the opposite effects of HS and CS on LAR clustering regulation. Heparin, a highly sulfated form of HS, or CS were applied to cells transfected with dimerization-uninducible pair of LARs (V5_LAR_LgBiT+Myc_LAR_SmBiT) and cells transfected with dimerization-inducible pair of LARs (V5_LAR_FRBLgBiT+Myc_LAR_FKBPSmBiT), in the presence or absence of rapamycin (20 nM). Analysis of the change in luminescence following chemicals application showed that, for the uninducible pair, regardless of rapamycin presence, heparin (40 mg ml^−1^) application significantly increased luminescence (Fig. 5A,B). For the inducible pair, the significant increase in luminescence following heparin (20 mg ml^−1^ or 40 mg ml^−1^) application was observed in the absence of rapamycin, but not in the presence of rapamycin when the dimerization of this pair was also induced by rapamycin (Fig. 5A,C). For CS application, the opposite effect was observed. Regardless of rapamycin presence, CS (40 mg ml^−1^) application to the uninducible pair significantly reduced luminescence (Fig. 5D,E). While the reduction in luminescence following CS application was not observed in inducible pair in the absence of rapamycin, in the case where rapamycin was co-applied, CS (10 mg ml^−1^, 20 mg ml^−1^, 40 mg ml^−1^) treatment significantly reduced the luminescence generated by rapamycin-induced dimerization (Fig. 5D,F). These results reconfirmed current knowledge that HS promotes and CS inhibits LAR dimerization or clustering.

**Fig. 6.**
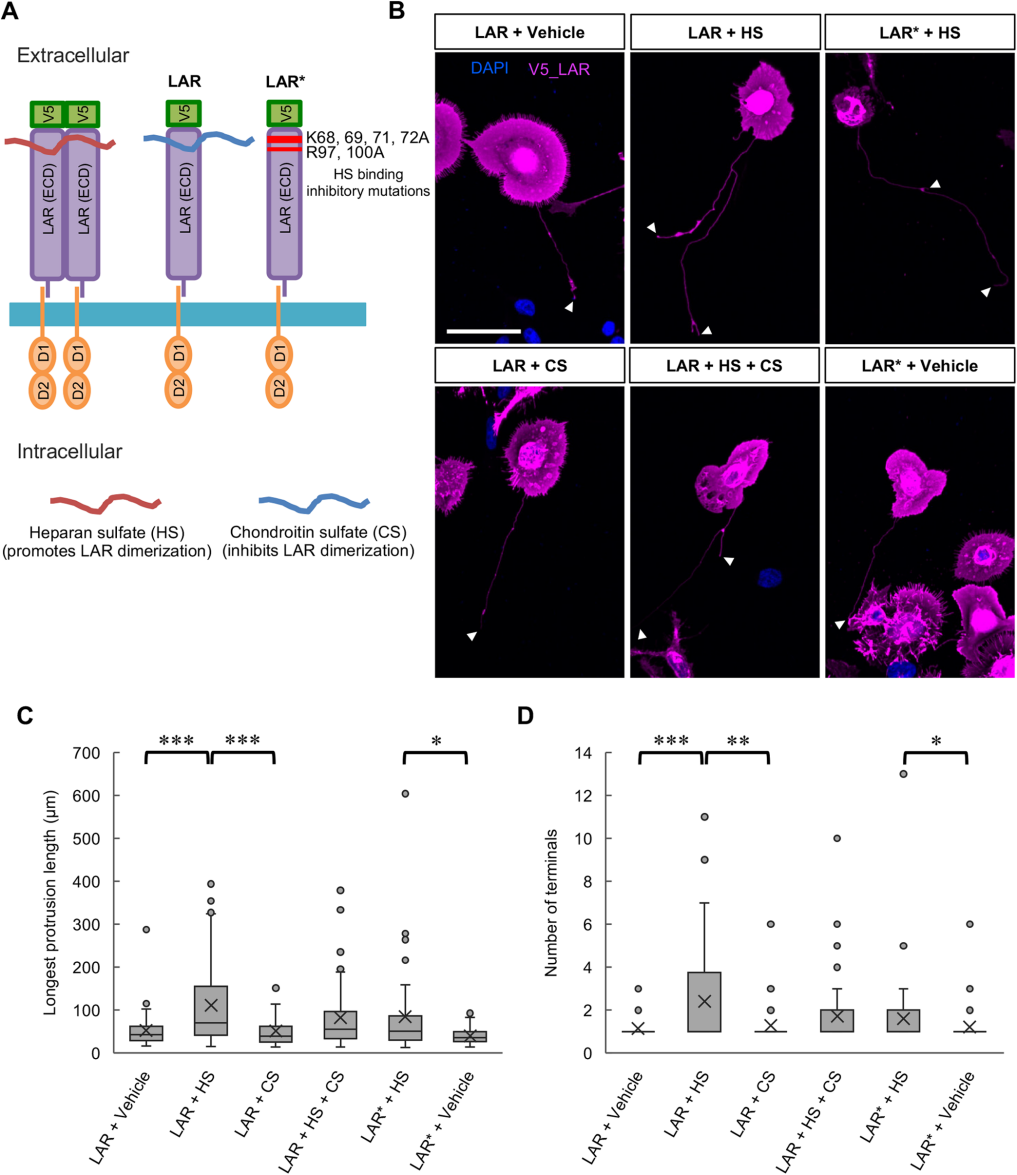
**LAR-induced protrusion formation was promoted in the presence of heparan sulfate.** (A) Schematic illustration of the LAR constructs used and of the LAR dimerization oppositely controlled by heparan sulfate and chondroitin sulfate. LAR*: LAR with K68A, K69A, K71A, K72A, R97A, and R100A mutations. ECD: extracellular domain. (B) Representative images from three independent trials showing the effects of heparan sulfate (HS) and chondroitin sulfate (CS) treatments on LAR- or mutated LAR*- induced protrusion formation. DAPI: blue, V5_LAR: magenta. Arrowheads: counted protrusion end tips (terminals). Scale bar: 40 µm. (C) Analytical results of the longest protrusion length. *N*=3 independent trials; total *n*=49, 66, 49, 55, 46, and 48 cells, respectively. *P<0.05 and ***P<0.001, nonparametric Wilcoxon test. In the box plot, the horizontal line within each box denotes the median value, and the x mark denotes the mean value. (D) Analytical results of the longest protrusion complexity. *N*=3 independent trials; total *n*=49, 66, 49, 55, 46, and 48 cells, respectively. *P<0.05, **P<0.01, and ***P<0.001, nonparametric Wilcoxon test. In the box plot, the horizontal line within each box denotes the median value, and the x mark denotes the mean value.

To investigate the effects of LAR-clustering regulation in protrusion formation, in the cell assay, the cells with at least one dominant long protrusion were analyzed to determine the protrusion length and branching complexity following the application of HS at 10 µg ml^−1^ or CS at 10 µg ml^−1^ to LAR-overexpressing HEK293T cells (Fig. 6A). Significant enhancement of the longest protrusion length and branching complexity was observed in LAR-overexpressing cells treated with HS compared to LAR-overexpressing cells treated with CS or vehicle (Fig. 6B,C,D). Unlike the effects seen in LAR-overexpressing cells, treatment of control (mock vector-expressing) cells with HS did not result in enhanced protrusion formation in terms of the longest protrusion length and branching complexity (Fig. S6).

To further confirm the opposing effects of HS and CS in the control of type IIa RPTP clustering, in this cell assay, CS was applied at a higher concentration (100 µg ml^−1^) together with HS (10 µg ml^−1^) to determine whether it could compete with and diminish the HS-mediated promotion of LAR-induced protrusion formation. Compared to the results in cells treated with HS only, in HS+CS-treated cells, treatment with 100 µg ml^−1^ CS was not enough to significantly reduce the effects of HS at 10 µg ml^−1^ on the promotion of the longest protrusion length (*P*=0.11 nonparametric Wilcoxon test) and branching complexity (*P*=0.12 nonparametric Wilcoxon test) (Fig. 6B,C,D).

Additionally, to test whether blockade of HS binding to LAR can diminish its promoting effects, a mutated construct of LAR with 6 mutations (K68, K69, K71, K72, R97, and R100 to alanine) in the Ig-like domains – well-known HS binding sites (Coles et al., 2011; Katagiri et al., 2018) (Fig. 6A) – was prepared and used to test the effects of HS application. The results showed that, compared to the wild-type LAR, LAR with these mutations was not able to completely inhibit the effects of HS on the longest formed protrusion length (*P*=0.05 nonparametric Wilcoxon test) or branching complexity (*P*=0.09 nonparametric Wilcoxon test) (Fig. 6B,C,D).

### Deletion of the phosphatase-related domains promoted LAR-induced protrusion formation, whereas introduction of phosphatase-inactive mutations into LAR showed no effects

**Fig. 7.**
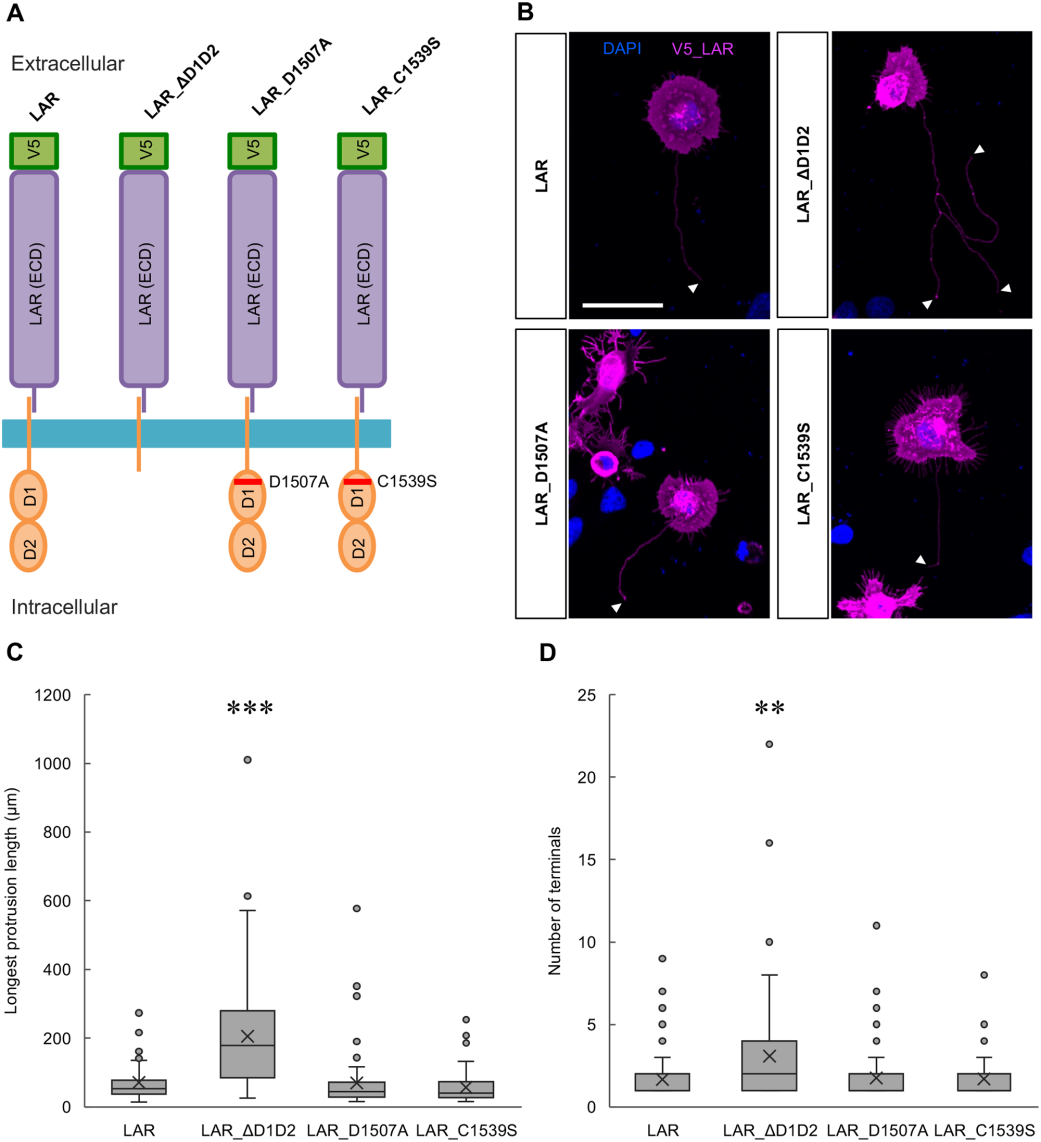
**Deletion of the phosphatase-related domains promoted LAR-induced protrusion formation, whereas phosphatase-inactive mutations showed no effects.** (A) Schematic illustration of the LAR constructs. LAR_ΔD1D2: LAR depleted of the phosphatase-related D1 and D2 domains. LAR_D1507A: LAR with #1507 aspartic acid mutated to alanine. LAR_C1539S: LAR with #1539 cysteine mutated to serine. ECD: extracellular domain. (B) Representative images from three independent trials on HEK293T cells expressing the respective LAR constructs. DAPI: blue, V5_LAR: magenta. Arrowheads: counted protrusion end tips (terminals). Scale bar: 40 µm. (C) Analytical results of the longest protrusion length. *N*=3 independent trials; total *n*=68, 109, 70, and 59 cells, respectively. ***P<0.0001, nonparametric Steel-Dwass test. In the box plot, the horizontal line within each box denotes the median value, and the x mark denotes the mean value. (D) Analytical results of the longest protrusion complexity. *N*=3 independent trials; total *n*=68, 109, 70, and 59 cells, respectively. **P<0.01, nonparametric Steel-Dwass test. In the box plot, the horizontal line within each box denotes the median value, and the x mark denotes the mean value.

Since the dimerization or clustering of LAR is proposed to regulate its phosphatase activity, we examined whether this enzymatic activity of LAR is essential for induced protrusion formation. For this purpose, we constructed a truncated form of LAR in which the intracellular phosphatase-related domains D1 and D2 were completely deleted (LAR_ΔD1D2) and mutated LAR forms in which key residues of the phosphatase D1 domain were mutated, which resulted in diminished enzymatic activity in accordance with previous literature (D1507A and C1539S) (Blanchetot et al., 2005; Dascenco et al., 2015; Tsujikawa et al., 2001; Xie et al., 2020) (Fig. 7A). To our surprise, the expression of these forms of LAR resulted in, in a sense, opposite outcomes. While the truncated form of LAR (LAR_ΔD1D2) promoted protrusion formation, resulting in significant increases in the longest protrusion length and branching complexity, the phosphatase-inactive mutants (LAR_D1507A and LAR_C1539S) did not show significant differences compared with the wild-type LAR (Fig. 7B,C,D). The membrane localization and protein expression of these LAR constructs were confirmed to be not significantly different (Fig. S7).

### The presence of heparan sulfate also promoted protrusion formation in the case of LAR with phosphatase-inactive mutations

**Fig. 8.**
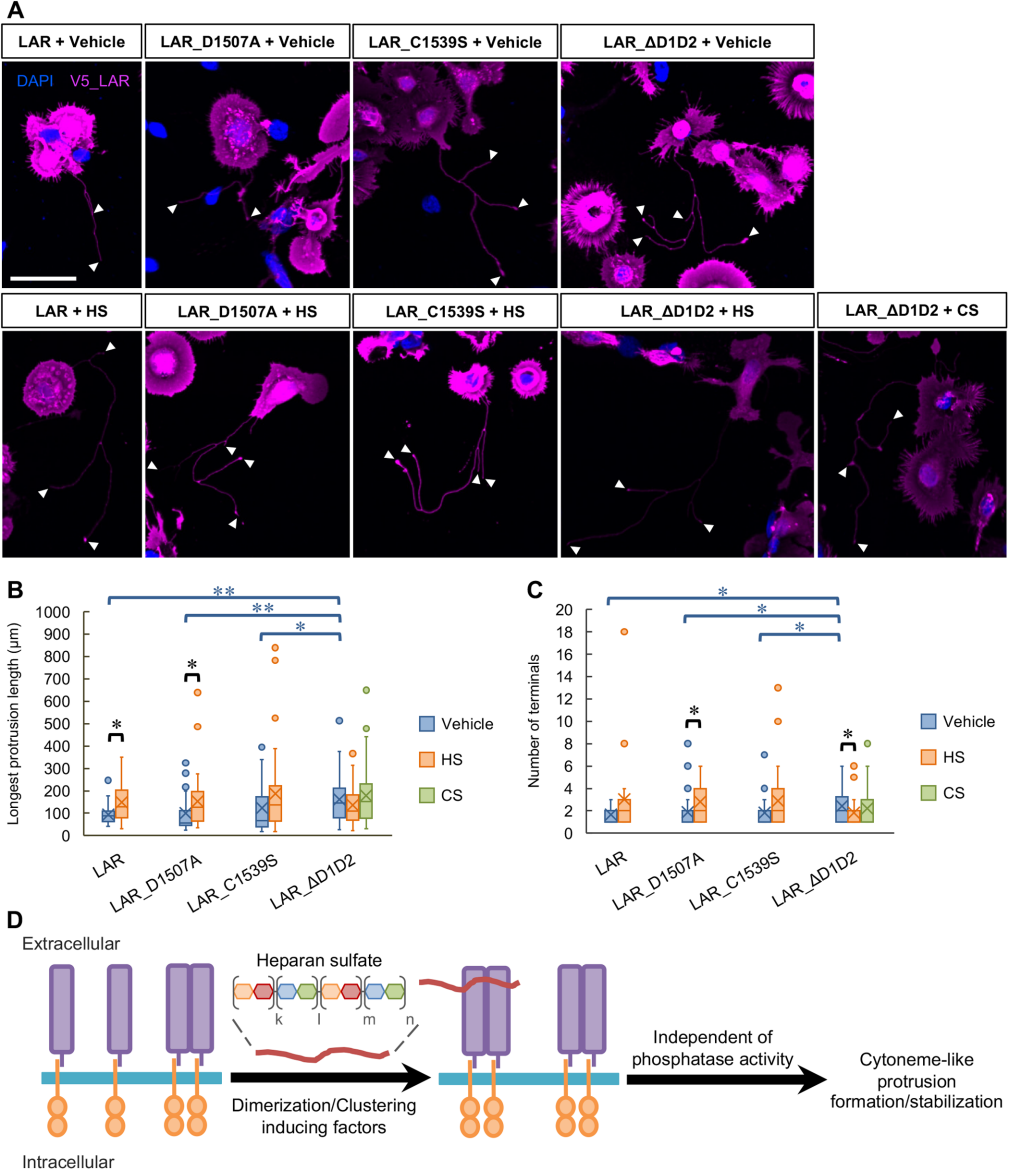
**The presence of heparan sulfate also promoted protrusion formation in the case of LAR with phosphatase-inactive mutations.** (A) Representative images from two independent trials showing the effects of heparan sulfate (HS) treatment on protrusion formation by LAR, LAR_ΔD1D2, LARs with phosphatase-inactive mutations: LAR_D1507A and LAR_C1539S expression; and of chondroitin sulfate (CS) treatment on LAR_ΔD1D2-induced protrusion formation. DAPI: blue, V5_LAR: magenta. Arrowheads: counted protrusion end tips (terminals). Scale bar: 40 µm. (B) Analytical results of the longest protrusion length. *N*=2 independent trials; total *n*=22, 23, 30, 35, 31, 37, 52, 42, and 43 cells, respectively. *P<0.05 and **P<0.01, nonparametric Wilcoxon test. In the box plot, the horizontal line within each box denotes the median value, and the x mark denotes the mean value. HS: heparan sulfate, CS: chondroitin sulfate. (C) Analytical results of the longest protrusion complexity. *N*=2 independent trials; total *n*=22, 23, 30, 35, 31, 37, 52, 42, and 43 cells, respectively. *P<0.05, nonparametric Wilcoxon test. In the box plot, the horizontal line within each box denotes the median value, and the x mark denotes the mean value. HS: heparan sulfate, CS: chondroitin sulfate. (D) Schematic illustration of LAR function in cytoneme-like protrusion formation. The ability of LAR to induce the formation of cytoneme-like protrusions is enhanced by its dimerization or clustering, which is promoted by extracellular factors such as heparan sulfate. This process, however, appears to be independent of LAR phosphatase activity.

To confirm that dimerization or clustering is truly important in promoting the protrusion formation, we applied HS to the cells transfected with LAR_ΔD1D2, LAR_D1507A, or LAR_C1539S (Fig. 8A,B,C). Compared to vehicle treatment, the application of HS (10 µg ml^−1^) promoted protrusion formation resulting into significantly longer and more branching protrusions in cells transfected with LAR_D1507A. In the case of LAR_C1539S, the increase in protrusion length and branching complexity was observed following HS treatment, however this was short of significancy (for the longest protrusion length *P*=0.096, for the number of terminals *P*=0.058 nonparametric Wilcoxon test). Differ to the case of LARs with phosphatase-inactive mutations, HS did not promote protrusion formation in cells transfected with LAR_ΔD1D2. Suspecting that there is a difference in basal dimerization or clustering state among these LAR constructs, with LAR_ΔD1D2 more prone to dimerize or cluster, we applied CS (10 µg ml^−1^) to cells expressing LAR_ΔD1D2 to examine whether CS could inhibit protrusion formation in the case of LAR_ΔD1D2 expression. The result, however, showed no significant changes in protrusion length and number of terminals (Fig. 8A,B,C) comparing vehicle or HS to CS treatments. The significant difference in terms of the longest protrusion length and the number of terminals observed in cells expressing LAR_ΔD1D2 compared to cells expressing LAR, LAR_D1507A, or LAR_C1539S was reconfirmed based on the vehicle treated samples analysis (Fig. 8A,B,C). From these results, we can conclude that it is the dimerization or clustering of LAR, induced by factors like HS, that promotes protrusion formation and this process appears to be independent of the LAR's phosphatase activity (Fig. 8D).

## DISCUSSION

LAR overexpression in HEK293T cells induced the formation of membrane protrusions (Fig. 1B,C) that possessed characteristic features of cytonemes: they were composed mainly of actin (Fig. 2; Figs S1-S4) and they may function as signaling conduits (Fig. S3). However, further evidence is required to define these formed protrusions as authentic cytonemes, especially regarding their role in the transport of signaling components or their involvement in the various signaling processes, then we here use the term ‘cytoneme-like’ instead. Compared to previously reported cytonemes, which have been described as fairly straight, the LAR-induced protrusions displayed a more complex morphology with branches (Fig. 1B). This suggests the importance of LAR in inducing the formation of protrusions capable of reaching different targets simultaneously to form a cellular communication network in the *in vivo* system. In addition, the total length of the LAR-induced longest protrusion in a cell was also longer (some protrusions exceeding 200 µm) than the previously reported cytonemes in *Drosophila* and the cytoneme-like protrusions induced by expression of the stem cell markers Lgr4 and Lgr5 in the HEK cell line (up to 80 µm) (González-Méndez et al., 2019). These results suggest that LAR exhibits a novel function in cytoneme-like protrusion formation by playing important roles, possibly in all of the processes: initiation, elongation, and branching.

Although it was not directly addressed in this study, the importance of LAR for the stabilization of the formed thin protrusions cannot be ignored. According to previous reports about cytonemes and cytoneme-like protrusions, these membrane structures are very fragile and sensitive to fixatives. They are very easily damaged and severed in the processes of fixation and immunocytochemical or immunohistochemical assays. The 4% PFA commonly used for fixation is not enough to protect these structures; live imaging is proposed to be a much more suitable method for their study (Snyder et al., 2015). Regarding this issue, the LAR-induced protrusions appeared to be relatively stably preserved with the use of 4% sucrose, 4% PFA in PBS for fixation (see Materials and Methods). With this method, although multiple PBS washes in the immunostaining procedures caused some observable segmentation in some long protrusions, they did not cause total washout or complete destruction of these structures.

To address the importance of LAR dimerization or clustering for the function of LAR in protrusion formation, we established a new rapamycin-induced LAR dimerization system (Fig. 3) and showed that the induced LAR dimerization promoted the formation of longer and more branched protrusions (Fig. 4). However, it is important to note that there are many possible mechanisms by which RPTPs, including LAR, actually form dimers or clusters under the regulation of various extracellular and intracellular factors (Coles et al., 2014; Johnson and Van Vactor, 2003; Um et al., 2014). Here, we showed that the fusion of FRB and FKBP to the C-terminus of LAR, and its intracellular dimerization induction by rapamycin was enough to assure the cascade triggered by this clustering, ultimately resulting in the morphological changes in the induced protrusions. Additionally, this induced dimerization state of LAR was reduced by CS treatment and was not further promoted by heparin (a highly sulfated form of HS) application (Fig. 5), suggesting that it resembles the LAR's endogenous dimerization state. Although, due to the potential of rapamycin to induce very high dimerization state of the inducible pair, the additive or synergic effects of HS (heparin) and rapamycin co-application might have been obscured, the detected canceling effects upon co-application of CS and rapamycin indicates a link between the extra- and intracellular domains of LAR in its clustering regulation.

Confirming the opposite effects of HS and CS in the regulation of LAR dimerization or clustering (Fig. 5), we further examined the importance of LAR dimer or cluster regulation in protrusion formation by applying these naturally existing extracellular matrix components to the cell cultures. The results strongly emphasized the importance of LAR clustering in promoting the formation of both longer and more branched protrusions (Fig. 6). The significant increase in both the longest protrusion length and complexity upon HS application compared to vehicle treatment in the cells expressing the mutated form of LAR – in which residues crucial for HS and CS binding in the well-known recognition sites within the Ig-like domains were mutated – (Fig. 6) suggested the existence of other binding sites for HS in LAR. This possibility is also supported by the fact that the application of a higher CS concentration, although did show reducing effects, was not enough to entirely suppress the effects of HS on LAR-induced protrusion formation (Fig. 6). Indeed, in PTPσ – another member of the type IIa RPTPs – novel heparin binding region located extracellularly in the vicinity of the transmembrane domain has been reported in addition to the shared binding sites for HS and CS in the Ig-like domains (Katagiri et al., 2018). With regard to LAR, a recent report has clarified that the human LAR fibronectin domains 5 to 8 can also bind heparin (Vilstrup et al., 2020). Together with the results yielded from the rapamycin-induced LAR dimerization system, these results suggest that further studies are required to elucidate the mechanism by which HS and/or CS and other extracellular and intracellular factors actually regulate the dimer or cluster formation of LAR as well as other RPTPs to control their function.

As previous reports have highlighted the relationship between dimerization or clustering of RPTPs (including LAR) and changes in their phosphatase activity (Ensslen-Craig and Brady-Kalnay, 2004; Johnson and Van Vactor, 2003; Nam et al., 1999; Xie et al., 2020), we examined whether interference with these enzymatic properties would affect LAR-induced cytoneme-like protrusion formation using a form of LAR lacking both phosphatase-related domains (D1 and D2 domains) and forms of LAR with mutations in the D1 domain that are suggested in the literature to interfere with enzymatic activity (Blanchetot et al., 2005; Dascenco et al., 2015; Tsujikawa et al., 2001; Xie et al., 2020). The results showed that the mutations inhibiting phosphatase activity did not affect LAR-induced protrusion formation, but the truncation of the phosphatase domains induced longer and more branched protrusions (Fig. 7) and that HS treatment promoted protrusion formation in the phosphatase-inactive LAR-expressing cells, but did not further promote the protrusion formation in cells expressing LAR lacking both phosphatase-related domains (Fig. 8), all suggesting that the functions of LAR in cytoneme-like protrusion formation were independent of its enzymatic activity and emphasizing the importance of LAR dimerization or clustering for its function. Regarding the enzymatic activity, indeed, a previous report has mentioned the ability of *Drosophila* LAR (DLAR) to function in a phosphatase-independent manner in the regulation of R7 photoreceptor axon targeting (Hofmeyer and Treisman, 2009). As for the importance of LAR clustering, it is proposed that D1-D1 interaction is important for the stabilization of dimer formation upon ligand binding (Xie et al., 2020). Thus, the lack of D1 in the truncated mutant might interfere with the dimer stabilization compared to the wild-type or phosphatase-inactive mutants in the case where these molecules form dimers by the induction of ligand. However, this D1-D1 interaction is described to be weak and unstable (Xie et al., 2020). Therefore, it can be inferred that in regards to baseline dimerization state in the absence of ligand, the lack of D1-D1 interaction in the case of the truncated mutant does not create a significance difference between this mutant and the wild-type LAR or phosphatase-inactive mutants. The observed promotion of protrusion formation in the case of truncated form of LAR might be, instead, due to the change in its molecular shape itself. In fact, it is suggested that membrane curvature – an initiation step for protrusion formation – is in one way induced by the crowding of asymmetrically shaped membrane proteins (Ljubojevic et al., 2021; McMahon and Boucrot, 2015). In agreement with this hypothesis, similar to other type IIa RPTPs, LAR has an asymmetric shape with extracellular domains larger in size than intracellular domains (Coles et al., 2014, 2015; Pulido et al., 1995; Stoker, 1994). Differ to the D1 mutated form of LARs, the truncated form of LAR lacking the intracellular domains resulted into a further asymmetrically shaped LAR with only bulky, yet very flexible, extracellular domains that affected the receptor interaction (Coles et al., 2014, 2015). Similar to the crowding of other asymmetrically shaped membrane proteins that alters local scaffolding of membranes (McMahon and Boucrot, 2015), the formation of asymmetric LAR dimers or clusters could have facilitated membrane curvature, resulting in an increase in the number of branching sites that was detected as an increase in the number of terminals. However, how LAR dimerization or clustering facilitates cytoskeletal rearrangement to activate protrusion elongation remains to be clarified. As our observations were in the *in vitro* context, which has limitations in mimicking the 3D *in vivo* settings that the LAR induced protrusions function in, it is important for further *in vivo* evidence to be acquired to elucidate the biological significance of these protrusions’ formation precisely. Despite that, it is suggested from our results that LAR is essential in inducing the formation of protrusions with branches, which are capable of reaching different targets simultaneously to support the formation of cellular communication networks in *in vivo* system. It can also be inferred that LAR is playing crucial roles in possibly all the processes from protrusions initiation or branching formation to its elongation.

In this study, using the HEK293T cell line, we showed that LAR participates in the formation of specialized filopodia (cytoneme-like membrane protrusions), possibly by functioning in the processes of membrane protrusion initiation, elongation, and, more importantly, branching, through clustering regulation in response to various extracellular inducers (such as heparan sulfate) in a phosphatase-independent manner (Fig. 8D). These findings suggest the possible involvement of thin membrane protrusions in cell signaling that may exist in *in vivo* systems in which LAR is expressed.

## MATERIALS AND METHODS

### Cloning and plasmid construction

LAR cloning was performed using PCR-based strategies with cDNA extracted from postnatal day 3 mouse brains. The primers used are listed in Table S1. The cloned LAR sequence was inserted into the pCAGGS-5MCS-HA vector with the restriction enzymes NotI and NheI. The full-length LAR-V5 tagged construct was prepared by digestion of the pCAGGS-LAR-HA plasmid with the restriction enzymes NotI and NheI followed by insertion of the resulting sequence into the pCAGGS-5MCS-V5 vector. These constructs were the basis for the preparation of the various LAR-expressing plasmids used in this study.

For preparation of the pEB_PPTsp_V5_LAR or pEB_PPTsp_Myc_LAR constructs, pCAGGS-LAR-HA was used as a template for PCR using the primers listed in Table S1, and the resulting product (the LAR sequence) was introduced to the pEB_PPTsp_V5_5MCS and pEB_PPTsp_Myc_5MCS vectors using the restriction enzymes SalI and NotI. pEB_PPTsp_V5_5MCS and pEB_PPTsp_Myc_5MCS vectors were prepared by inserting the coding sequence for pre-protrypsin signal peptide (PPTsp) followed by the V5 or Myc tag into the KpnI-XhoI sites of pEBMulti-neo (Wako Pure Chemical Industries, Ltd., Osaka, Japan). The primers used are listed in Table S1.

For preparation of the truncated form of LAR lacking the D1 and D2 domains, the pEB_PPTsp_V5_LAR vector was used as a template for PCR using the primers listed in Table S1, and the resulting product (the LAR_ΔD1D2 sequence) was introduced into the pEB_PPTsp_V5 backbone vector using the restriction enzymes SalI and NotI.

Mutation insertion into the LAR was performed using PCR-based strategies in combination with restriction enzyme cutting and ligation and/or in-fusion cloning. In-Fusion cloning was performed using the In-Fusion^®^ HD Cloning Kit (Clontech Laboratories, Inc., Shiga, Japan) following instructions provided in the user manual.

LAR constructs with split domains of luciferase (LgBiT/SmBiT) were prepared using PCR-based strategies in accordance with the instructions provided by Promega Corporation (WI, USA). First, the pCAGGS-LAR-HA construct was used as a template, and the amplified sequence was inserted into the pFA CMV Flexi^®^ Vector (Promega Corporation). Next, the target sequence was cut and inserted into the pFC34 K LgBiT TK-Neo Flexi^®^ Vector or the pFC36K SmBiT TK-Neo Flexi^®^ Vector containing the HSV-TK promoter (Promega Corporation). The primer sequences are listed in Table S1. These constructs were originally created with the HSV-TK promoter; later, using suitable restriction enzymes, the LgBiT/SmBiT-fused LAR sequences were transferred to the pEB_PPTsp_V5_LAR vector under the control of the CAG promoter.

Constructs of LAR with FRBLgBiT/FKBPSmBiT sequences were prepared by transferring the LAR sequence from pFA CMV Flexi^®^ Vector_LAR (previously made) to the FRB-LgBiT control vector or FKBP-SmBiT control vector with the HSV-TK promoter (Promega Corporation) and then inserting a linker between LAR and FRB or between LAR and FKBP. The linker of the constructs used in this study was GGGGSGGGG. Later, the pEB_PPTsp_V5_LAR_FRBLgBiT and pEB_PPTsp_V5_LAR_FKBPSmBiT constructs (LAR expression under the control of the CAG promoter) were constructed using the pEB_PPTsp_V5_LAR vector as the backbone and suitable restriction enzymes for cutting and transferring LAR_FRBLgBiT or LAR_FKBPSmBiT from the pHSV-TK promoter vectors.

The pEB_PPTsp_Myc_LAR_SmBiT and pEB_PPTsp_Myc_LAR_FKBPSmBiT constructs were made using the pEB_PPTsp_Myc_LAR vector as the backbone to transfer the restriction enzyme-cut LAR_SmBiT or LAR_FKBPSmBiT from the previously made pEB_PPTsp_V5_LAR_SmBiT or pEB_PPTsp_V5_LAR_FKBPSmBiT.

All the LAR constructs used in this study are listed in Table S2. The LAR expression constructs coded for LAR with three Ig-like domains (Ig1, Ig2, and Ig3) and five fibronectin type III repeats (FN1, FN2, FN3, FN5, and FN8) on the extracellular side. These constructs of LAR contained three mini-exons: meA, meC, and meD. The amino acid sequence of the LARs used in this study are listed in Table S3.

### Cell line, cell culture and transfection methods

HEK293T cells [293T (ATCC CRL-3216)] (American Type Culture Collection, ATCC, VA, USA) were cultured in 1X Dulbecco's modified Eagle's medium (DMEM) (Nacalai Tesque, Kyoto, Japan) with 10% fetal bovine serum (Sigma-Aldrich, Munich, Germany or Nichirei Biosciences Inc., Tokyo, Japan), 100 U ml^−1^ penicillin (Meiji Seika Pharma Co., Ltd., Tokyo, Japan), and 100 μg ml^−1^ streptomycin (Sigma-Aldrich, Munich, Germany) at 37°C in a humified incubator with a 5% CO_2_ air supply. For cell line maintenance, HEK293T cells were cultured in 10 cm plastic dishes (Thermo Fisher Scientific, MA, USA). For live-cell confocal imaging, HEK293T cells were subcultured on four-well chambered glass coverslips (Matsunami Glass Ind., Ltd., Osaka, Japan) coated with 0.0033% poly-L-lysine, diluted from the 0.01% stock poly-L-lysine solution (Sigma-Aldrich). For cell counting and immunocytochemical and morphological analyses of protrusions or evaluation of LAR constructs’ membrane expression, HEK293T cells were subcultured on four-well chambered glass slides (Matsunami Glass Ind., Ltd., Osaka, Japan) coated with 0.0033% poly-L-lysine, diluted from the 0.01% stock poly-L-lysine solution (Sigma-Aldrich) or prepared by diluting Poly-L-lysine hydrobromide powder (Sigma-Aldrich) in distilled water. For electron microscopic observation, HEK293T cells were subcultured in 35 mm plastic dishes (Thermo Fisher Scientific). For the luciferase assay, HEK293T cells were subcultured in 96-well plastic-bottom plates (FALCON Corning, New York, NY, USA). For western blot, HEK293T cells were subcultured in 12-well plastic dishes (Thermo Fisher Scientific).

Cells were transfected using polyethylenimine (PEI, Polysciences, Inc., PA, USA) in accordance with the provided protocol. About three hours post transfection, the media were renewed to avoid cytotoxicity caused by the transfectant. This procedure was omitted in the case of transfection for the luciferase assay when 96-well plates were used.

### Immunocytochemistry

HEK293T cells were seeded at a concentration of 0.5∼1×10^4^ cells/well on four-well chambered glass slides and then transfected with plasmids at 48 h post plating. The culture media were renewed about 3 h post transfection, and the culture was performed for a total of 48 h post transfection until fixation (the cells remained fairly sparsely distributed).

In the experiments in which heparan sulfate (HS) and/or chondroitin sulfate (CS) or rapamycin was used, application was performed 24 h post transfection, and the treatment was continued for 24 h until fixation. The application concentrations were as follows: HS 10 µg ml^−1^, CS 10 µg ml^−1^, HS 10 µg ml^−1^+CS 100 µg ml^−1^, and rapamycin 100 nM. The applied HS and CS concentrations were selected as above so that cells remained fairly healthy following the 24-h incubation. The induced dimerization detected as change in luminescence in the luciferase assay, in which rapamycin effect was recorded for 1 h, showed no significant difference between rapamycin treatment of 20 nM and 100 nM. Although we did not perform the cell assay with 20 nM rapamycin, to assure that an efficient amount of rapamycin is provided during the 24-h incubation until fixation, we applied the 100 nM concentration. Heparan sulfate (GAG-HS01) was obtained from Iduron Ltd. (Cheshire, UK) and chondroitin sulfate sodium salt from shark cartilage (C4384) was obtained from Sigma-Aldrich. Rapamycin was obtained from Wako Pure Chemical Industries, Ltd. (Osaka, Japan).

The cells were fixed in 4% PFA, 4% sucrose in PBS for 15 min and then subjected to three PBS washes. Then, permeabilization was performed with 0.25% Triton X-100 for 5 min and was followed by three PBS washes. Blocking using 10% normal goat serum in PBS was performed for 1 h at room temperature. Next, staining with primary antibodies in 2% normal goat serum in PBS was performed for 1 h at room temperature and was followed by three PBS washes. Then, the samples were incubated with secondary antibodies in 2% normal goat serum in PBS for 30 min at room temperature, washed twice with PBS, stained for 5 min with DAPI in PBS, and washed twice more in PBS before being mounted using the fluorescence mounting medium Fluoromount (Diagnostic BioSystems, Inc., CA, USA).

The primary antibodies and dilution ratios used are listed in Table S4.

The secondary antibodies and dilution ratios used are listed in Table S5.

### Investigation of the cytoskeletal components of LAR-induced protrusions

Immunocytochemical staining against F-actin (using fluorescently tagged phalloidin) or against alpha-tubulin were performed using HEK293T cells transfected with V5_LAR or V5_5MCS (mock) and the Lyn11-EGFP expression vectors. Optical images of the cells were taken using an FV3000 confocal microscope (Olympus, Tokyo, Japan) with a 60X objective lens. The acquired fluorescence images were processed using IMARIS (Bitplane, Belfast, UK).

Electron microscopic image acquisition was performed using HEK293T cells transfected with V5_LAR or V5_LAR_ΔD1D2 and the Lyn11-EGFP expression vectors. HEK293T cells were seeded at a concentration of 3.5×10^4^ cells/dish in 35 mm plastic dishes and then transfected with plasmids at 24 h post plating. The culture media were renewed 3 h post transfection, and the cultures were continued for a total of 48 h post transfection until image acquisition. First, optical images of the living cells were taken using a BZ9000 light microscope (Keyence, Osaka, Japan) for the purpose of target cell identification. The selected cells were labeled with the green fluorescence of Lyn11-EGFP, that was co-transfected with LAR, and displayed morphology resulting from the LAR-expression: having multiple short protrusions or having a dominantly thin and long protrusion. Soon after, the cells were fixed with 2% PFA and 2.5% glutaraldehyde in 0.1 M PB overnight at 4°C. The fixed cultures were rinsed with 0.1 M PB, immersed in 1% osmium tetroxide in 0.1 M PB for 1 h on ice and subsequently stained with 0.5% uranyl acetate in distilled water overnight at 4°C. The samples were then dehydrated in increasing concentrations of ethanol (65%, 75%, 85%, 95% and 100%) at room temperature, with a 5 min incubation time in each ethanol concentration. They were next dehydrated three times with anhydrous ethanol processed with a molecular sieve and twice with 2-hydroxypropyl methacrylate at room temperature for 20 min per incubation. Subsequently, the samples were immersed in a 1:1 solution of 2-hydroxypropyl methacrylate:epoxy resins overnight at room temperature and embedded using epoxy resins by inverting a BEEM capsule-like block, which was polymerized with the resin, on the target area. After polymerization, ultrathin sections were cut using a Reichert-Nissei Ultracut N microtome (Leica, Wetzlar, Germany), observed and image-acquired using an H-7650 transmission electron microscope (Hitachi, Tokyo, Japan) with uranyl acetate and lead citrate counterstains.

### Live-cell time-lapse imaging

HEK293T cells were seeded at a concentration of 0.5×10^4^ cells/well on four-well chambered glass coverslips and then transfected with plasmids (co-transfection of V5_LAR and the Lyn11-EGFP expression vectors) at 48 h post plating. The culture media were renewed 3 h post transfection. Then, 40 h post transfection, vehicle (DMSO 0.1%; Wako Pure Chemical Industries, Ltd., Osaka, Japan) or 2 µM cytochalasin D (FUJIFILM Wako Pure Chemical Corporation, Osaka, Japan) or 2 µM nocodazole (FUJIFILM Wako Pure Chemical Corporation, Osaka, Japan) was applied. At 8 h post application, the selected cells (labeled with the green fluorescence of Lyn11-EGFP, that was co-transfected with LAR, and displayed morphology resulting from the LAR-expression: having a dominantly thin and long protrusion) were live-imaged using a SpinSR10 spinning disk confocal microscope (Olympus, Tokyo, Japan) with a humidified, heated stage (37°C) supplied with 5% CO_2_. Imaging was performed for 1 h and was followed by washout of the chemicals with two washes in DMEM-10% FBS/PS. Imaging of the same cells was performed for another 2.5 h. Images were acquired every 5 min with a 40X objective lens.

The acquired z-stack time-lapse images were rendered in three dimensions and processed before tracing and tracking of the longest formed protrusion were performed for each imaged cell. All of these analytical processes were semi-automatically performed using IMARIS (Bitplane, Belfast, UK). For tracing and tracking, all the images were processed with a gamma correction of 1.5. The display images in related figures and movies have no gamma correction (gamma=1.0).

### Cell-count analyses

Cell-count analyses were performed for HEK293T cells transfected with V5_LAR or V5_5MCS (mock) and the Lyn11-EGFP (for visualization of the membrane protrusions) expression vectors. Immunocytochemical staining against V5 and EGFP as well as DAPI staining were performed. Optical z-stack images of the cells were taken using a SpinSR10 spinning disk confocal microscope (Olympus, Tokyo, Japan) with a 20X objective lens. The acquired images were processed using Cell Sens (Olympus, Tokyo, Japan) (with the same parameters for all images, including a gamma correction of 1.5 for all channels), and counting of cells with protrusions was performed manually using Photoshop (Adobe, Inc., CA, USA). Cells fully observable within the acquired images and double-labeled with anti-V5 and anti-EGFP were counted, and their protrusions were analyzed based on the Lyn11-EGFP immunocytochemical staining signals. We did not exclude the round, small cells (those without protrusions) that might be unhealthy cells resulted from the culture process or from immunocytochemistry process. Therefore, within the total counted cells there were cells without protrusion(s). In addition, to acquire a broader region of interest within each image for cell counting, as described above, we used the 20X objective lens, this might have obscured the very small protrusions that are visible at higher magnifications. Following counting, the percentage of cells with protrusion of different length were calculated for each image, then the average percentage of these cells for each trial was calculated. Statistical test was performed using average percentage of cells obtained from three independent trials, comparing V5_LAR and V5_5MCS (mock) expression samples. The representative images in Fig. 1 were acquired using an FV3000 confocal microscope (Olympus, Tokyo, Japan) and processed without gamma correction (gamma=1.0) using FV31S-SW (Olympus). The DIC images were processed using ImageJ (National Institutes of Health, MA, USA).

### Morphological analyses of protrusions

In the HS/CS application experiments conducted on HEK293T cells expressing V5_LAR or the mutated version V5_LAR* (K68, 69, 71, 72A and R97, 100A) or truncated LAR lacking the D1 and D2 domains (V5_LAR_ΔD1D2) or phosphatase-inactive LAR (V5_LAR_D1507A or V5_LAR_C1539S), immunocytochemical staining against V5 and DAPI staining were performed. In HS application experiments using HEK293T cells transfected with V5_LAR or V5_5MCS (mock) and the Lyn11-EGFP (for visualization of the membrane protrusions) expression vectors, immunocytochemical staining against V5 and EGFP and DAPI staining were performed. Optical z-stack images of the cells were taken using a SpinSR10 spinning disk confocal microscope (Olympus, Tokyo, Japan) with a 40X objective lens. The acquired images were processed (with the same parameters for all images of the same experiment type and a gamma correction of 2.0 for all channels), and the induced longest protrusions were semi-automatically traced for total length and complexity evaluation (tracing analysis) based on the immunocytochemical staining signals of V5 or EGFP (in the experiment where a mock vector was used) using IMARIS (Bitplane, Belfast, UK). The representative images in related figures were processed with no gamma correction (gamma=1.0).

In experiments in which rapamycin (100 nM) was applied to induce LAR dimerization, HEK293T cells were transfected with V5_LAR_FRBLgBiT/Myc_LAR_FKBPSmBiT-expressing vectors, control (1) V5_LAR_LgBiT/Myc_LAR_SmBiT-expressing vectors, control (2) V5_LAR_FRBLgBiT-expressing vectors, or control (3) Lyn11-EGFP-expressing vectors. Immunocytochemical staining against V5 and Myc or only V5 [in the case of control (2)] or against EGFP [in the case of control (3)] was performed together with DAPI staining. Optical z-stack images of the cells were taken using a SpinSR10 spinning disk confocal microscope (Olympus, Tokyo, Japan) with a 40X objective lens. The acquired images were processed (with the same parameters for all images and gamma corrections of 2.0 for channel 405 and 1.5 for channels 488 and 561), and the induced longest protrusions were semi-automatically traced for total length and complexity evaluation (tracing analysis) based on the immunocytochemical staining signals of V5 using IMARIS (Bitplane, Belfast, UK). Because cells with long protrusions were rarely seen, tracing analysis was not performed for control (3), and the acquired images for control (3) were single plane images. The representative images in related figures were processed with no gamma correction (gamma=1.0).

In the experiment to assess the involvement of LAR phosphatase activity, HEK293T cells were transfected with expression vectors for LAR, truncated LAR lacking the D1 and D2 domains (V5_LAR_ΔD1D2) or phosphatase-inactive LAR (V5_LAR_D1507A or V5_LAR_C1539S) together with a Lyn11-EGFP-expressing construct. Immunocytochemical staining against V5 and EGFP as well as DAPI staining were performed. Optical z-stack images of the cells were taken using a SpinSR10 spinning disk confocal microscope (Olympus, Tokyo, Japan) with a 40X objective lens. The acquired images were processed (with the same parameters for all images and gamma corrections of 2.0 for channels 405 and 561 and 1.5 for channel 488), and the induced longest protrusions were semi-automatically traced for total length and complexity evaluation (tracing analysis) based on the immunocytochemical staining signals of V5 using IMARIS (Bitplane, Belfast, UK). The representative images in related figures were processed with no gamma correction (gamma=1.0).

Tracing analysis was performed for the longest protrusion formed in a cell. Branches of less than 1 µm in length, branches that originate from branching points located within 10 µm from the initiation point in the cell soma, and branches that originate from branching points located within 20 µm from the ending tips were excluded from the tracing.

### Rapamycin-induced LAR dimerization detection using a split luciferase assay

A split luciferase assay was conducted in accordance with the protocol provided by Promega Corporation (WI, USA). HEK293T cells were transfected with V5_LAR_FRBLgBiT/Myc_LAR_FKBPSmBiT-expressing plasmids or control V5_LAR_LgBiT/Myc_LAR_SmBiT-expressing plasmids so that the total DNA amount was 100 ng/well. After transfection, the cells were cultured at 37°C under 5% CO_2_ for 20∼24 h. Then, before application of the substrates, the culture media were exchanged with Opti-MEM^®^ I (1X) (Gibco^®^ by Life Technologies^TM^, Thermo Fisher Scientific) and incubated at ambient temperature for 10 min. The transfected wells were recorded for at least 5 min before substrate addition to assess the basal status. Luciferase substrate (Promega Corporation) was applied for 5 min before the generated luminescence was recorded for 40 counts. Then, vehicle or rapamycin (20 nM) (Wako Pure Chemical Industries, Ltd., Osaka, Japan) was applied, and the subsequent changes in luminescence were recorded for 61 counts. The interval between counts was 1 min. The whole process was performed at ambient temperature. Luminescence was detected using a Centro XS3 LB960 High Sensitivity Microplate Luminometer (Berthold Technologies, Bad Wildbad, Germany).

### Heparin or CS application in combination with rapamycin-induced LAR dimerization detection using the split luciferase assay

HEK293T cells were transfected with rapamycin-dimerization inducible pair (V5_LAR_FRBLgBiT/Myc_LAR_FKBPSmBiT) or rapamycin-dimerization uninducible pair (V5_LAR_LgBiT/Myc_LAR_SmBiT) so that the total DNA amount was 100 ng/well. After transfection, the cells were cultured at 37°C under 5% CO_2_ for about 22∼26 h. Then, before application of the substrates, the culture media were exchanged with Opti-MEM^®^ I (1X) and incubated at ambient temperature for 10 min. The transfected wells were recorded for at least 5 min before substrate addition to assess the basal status. Luciferase substrate was applied for 5 min before the generated luminescence was recorded for 30 counts. Then, vehicle or rapamycin (20 nM) together with heparin [0 mg ml^−1^ (vehicle), 10 mg ml^−1^, 20 mg ml^−1^, 40 mg ml^−1^] (Heparin sodium salt Nacalai Tesque, catalogue number 17513-54) or CS [0 mg ml^−1^ (vehicle), 10 mg ml^−1^, 20 mg ml^−1^, 40 mg ml^−1^] (Sigma-Aldrich) were applied, and the subsequent changes in luminescence were recorded for 121 counts. The interval between counts was 1 min. The whole process was performed at ambient temperature. Luminescence was detected using a Centro XS3 LB960 High Sensitivity Microplate Luminometer.

### Evaluation of the LAR constructs’ cell membrane expression

HEK293T cells, seeded at a concentration of 0.5x10^5^ cells/well on four-well chambered glass slides, were transfected with respective LAR constructs: V5_LAR, V5_LAR* (K68, 69, 71, 72A and R97, 100A), V5_LAR_ΔD1D2, V5_LAR_D1507A, V5_LAR_C1539S, V5_LAR_FRBLgBiT, V5_LAR_LgBiT, Myc_LAR_FKBPSmBiT, Myc_LAR_SmBiT. Then, immunocytochemical staining against V5 or Myc and DAPI staining were performed without detergent (Triton X-100) treatment (details of the procedure are described in the Immunocytochemistry section, omitting the detergent application and the following three PBS washes). Optical single plane images of the cells were taken using an FV3000 confocal microscope (Olympus, Tokyo, Japan) with a 100X objective lens. The acquired fluorescence images were analyzed using ImageJ (National Institutes of Health, MD, USA) with no gamma correction (gamma=1.0) to evaluate membrane expression of the LAR constructs, by measuring fluorescence signals detected in membrane, normalized to the signals detected in cytosol, and compare among the different constructs. The representative images in related figure were processed using IMARIS (Bitplane, Belfast, UK) with no gamma correction (gamma=1.0).

### Western blotting

HEK293T cells subcultured in 12-well plate were transfected with respective LAR constructs: V5_LAR, V5_LAR* (K68, 69, 71, 72A and R97, 100A), V5_LAR_ΔD1D2, V5_LAR_D1507A, V5_LAR_C1539S, V5_LAR_FRBLgBiT, V5_LAR_LgBiT, Myc_LAR_FKBPSmBiT, Myc_LAR_SmBiT, so that total amount of DNA was 1 µg/well. The culture media were renewed about 3 h post transfection, and the culture was performed for a total of about 48 h post transfection until cell collection. Each cell sample (well) underwent PBS wash (500 µl), followed by treatment of lysis buffer (150 mM NaCl, 25 mM Tris-HCl pH 7.5, 1 mM EDTA, 1% NP-40) (300 µl) for 15 min, before being collected and centrifuged at 15,000 rpm for 10 min at 4°C. Bradford method was used to measure protein concentration within each sample before these cell lysates were treated with sample buffer at 95°C for 3 min (the final protein concentration was 1 µg µl^−1^ for each sample) followed by separation of proteins by SDS-PAGE and their transfer to polyvinylidene difluoride (PVDF) membrane (Immobilon Transfer Membranes; EMD Millipore/Merck KGaA, Darmstadt, Germany). The membranes were blocked with 5% skim milk in TBST (0.05% Tween-20, 50 mM Tris-HCl, 138 mM NaCl, 2.7 mM KCl) at room temperature for at least 1 h, then were incubated with antibodies. Antibodies were washed off by shaking membranes in TBST three times for 15 min each time. Detection was done using Luminata Forte Western HRP substrate (EMD Millipore/Merck KGaA, Darmstadt, Germany). Images were captured with LAS-3000 mini (FUJIFILM, Tokyo, Japan) and the band intensity was analyzed using Multi Gauge V3.0 (FUJIFILM, Tokyo, Japan).

### Statistical analyses

In bar graphs, the data are presented as the mean±s.e.m., and the dots denote average observation values from each experimental trial. For line graphs, the dots represent observation values. In box plots, the horizontal line within each box denotes the median value, and the x mark denotes the mean value; the boxes extend from the 25th to the 75th percentile of each group's distribution of values, and the vertical extending lines (whiskers) present the most extreme values within 1.5 interquartile range of the 25th and 75th percentile of each group (adjacent values). The dots denote observations outside the range of adjacent values. All analyzed data were included in statistical analyses, without any exclusion. Statistically significant differences between two groups were calculated using Student's *t*-test or nonparametric Wilcoxon test. Differences between each pair in multiple-group analyses were calculated using nonparametric Wilcoxon test or nonparametric Steel-Dwass test. Differences between groups comparing with a control group were calculated using nonparametric Steel test. All statistical analyses were performed using JMP Pro 14 (SAS, NC, USA) with the chosen significance level α=0.05.

## Supplementary Material

Supplementary information
