# Supplementary material for: Cytoneme-like protrusion formation induced by LAR is promoted by receptor dimerization
Source: Biol Open. 2022 Jul 25;11(7):bio059024. doi: 10.1242/bio.059024 (PMC9346286; doi:10.1242/bio.059024)
Supplement: Supplementary information [file biolopen-11-059024-s1.pdf]

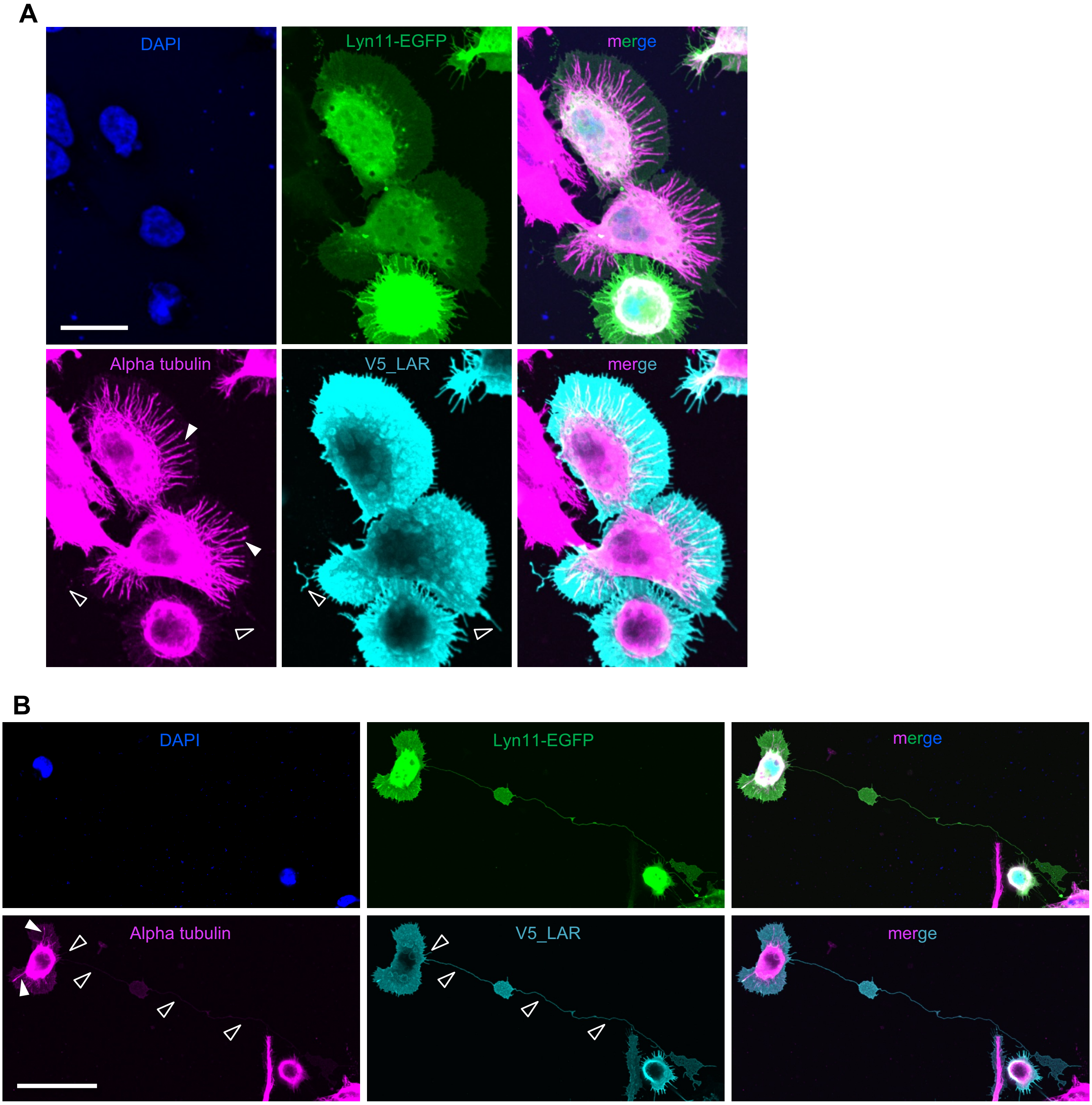

Fig. S1. LAR-induced protrusions showed very weak stain for alpha tubulin

(A) Representative images of alpha tubulin staining from three independent trials showing cells with short protrusions. DAPI: blue, Lyn11-EGFP: green, alpha tubulin: magenta, V5_LAR: cyan. In the alpha tubulin panel, the arrowheads point to clear signals within the cell soma and open arrowheads point to the very weak signal in short protrusions. In the V5_LAR panel, open arrowheads point to the respective sites indicated in the alpha tubulin panel. Scale bar: 20 µm.

(B) Representative images of alpha tubulin staining from three independent trials showing a cell with dominantly long protrusion. DAPI: blue, Lyn11-EGFP: green, alpha tubulin: magenta, V5_LAR: cyan. In the alpha tubulin panel, the arrowheads point to clear signals within the cell soma and open arrowheads point to the very weak signal in both the short and the long protrusions of the cell. In the V5_LAR panel, open arrowheads point to the respective sites indicated in the alpha tubulin panel. Scale bar: 40 µm.

Biology Open • Supplementary information

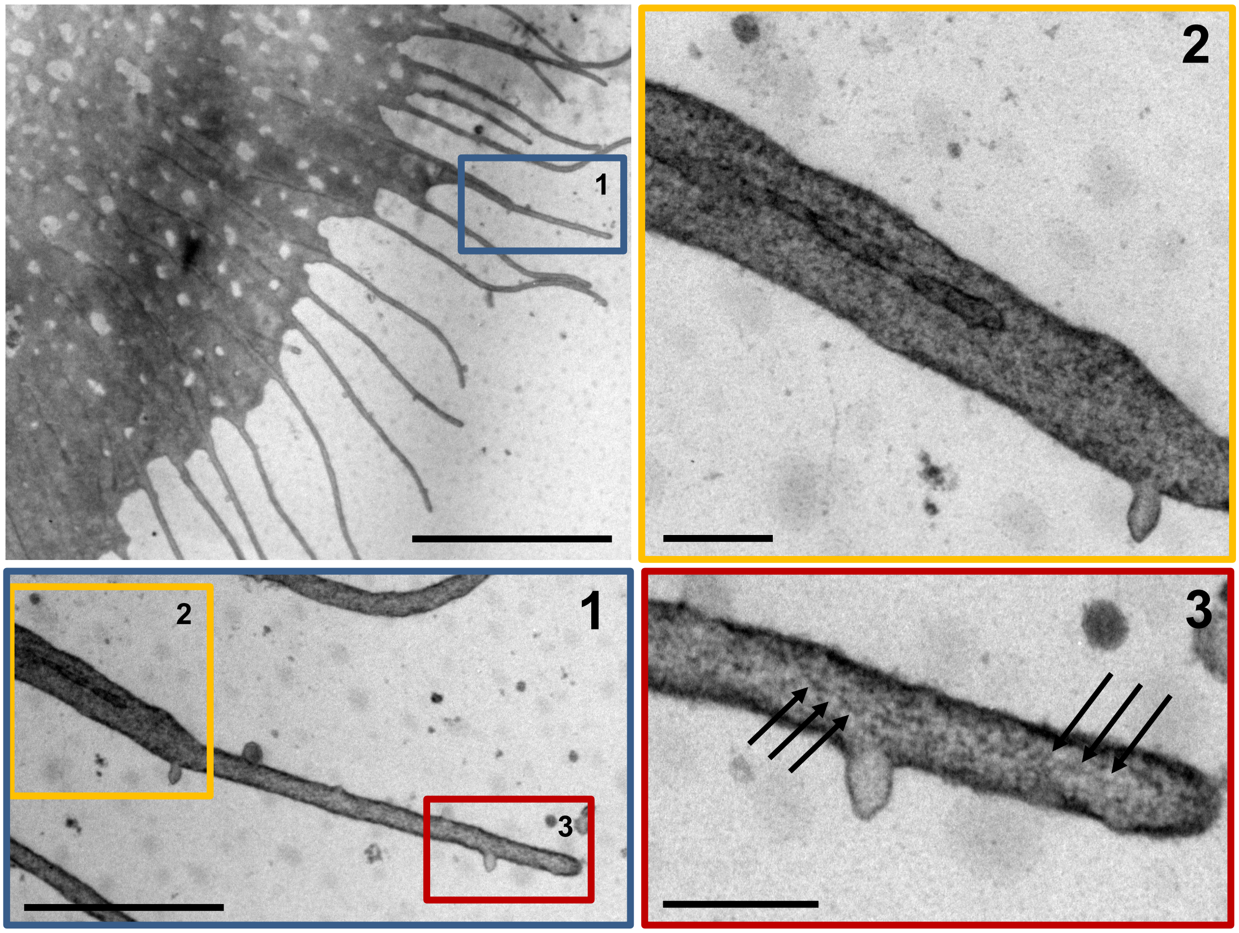

Fig. S2. Observed short protrusion contained actin

Electron microscopic images of a LAR and Lyn11-EGFP co-transfected cell with short protrusions showing the presence of microtubule at the base and actin fibers within a short protrusion. Inset 1 shows a short protrusion with microtubule at the base (inset 2) and actin fibers within (inset 3). Arrows point to the actin fibers. Scale bars: cell soma image: 4 μm, inset 1: 1 μm, inset 2: 200 nm, and inset 3: 200 nm.

Biology Open • Supplementary information

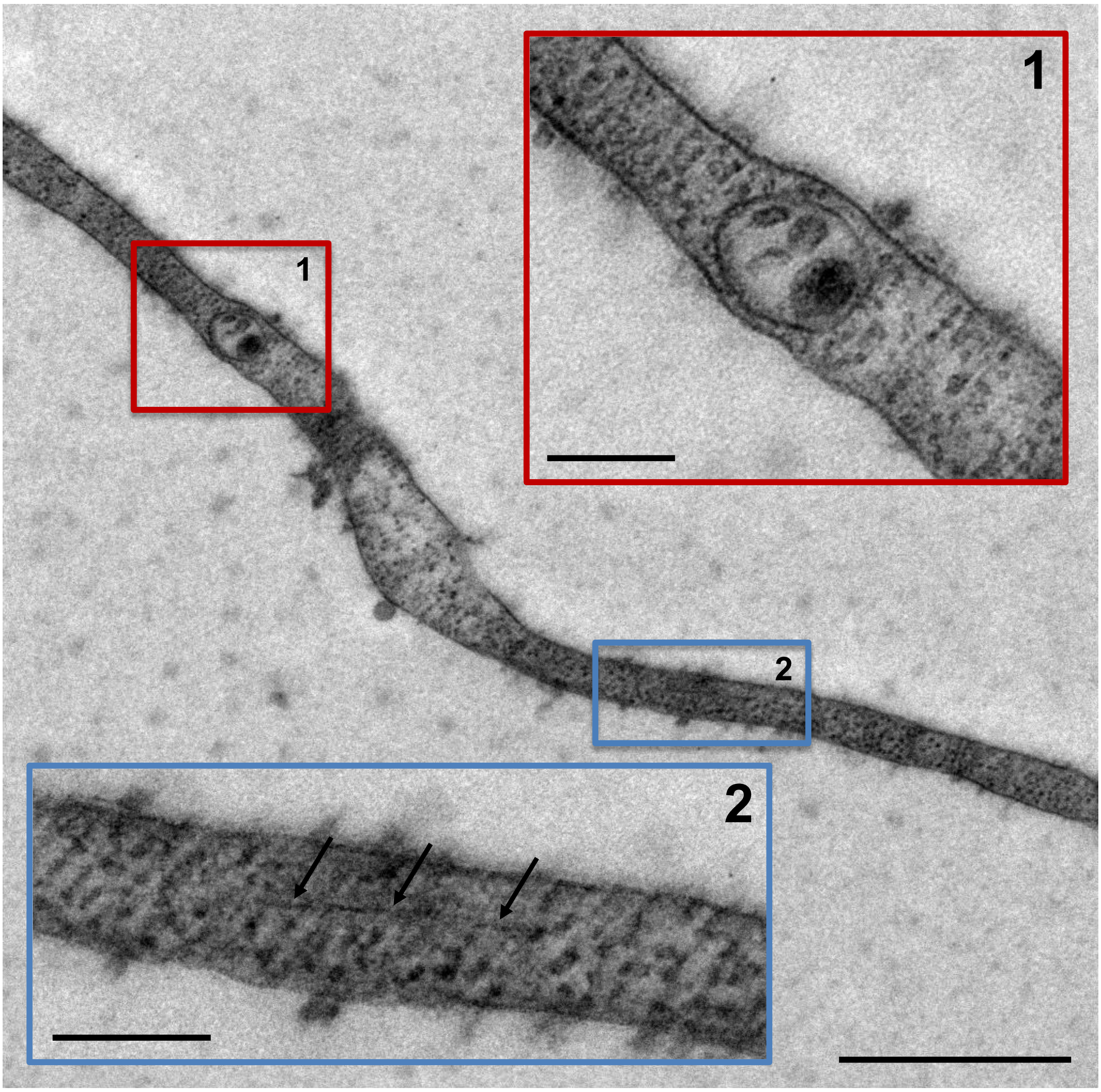

Fig. S3. Observed long protrusion contained actin and macrovesicle

Electron microscopic images of a segment of the long protrusion of a LAR_ ΔD1D2 and Lyn11-EGFP co-transfected cell showing the presence of actin fiber and macrovesicle within. Inset 1: magnified protrusion area containing macrovesicle. Inset 2: magnified protrusion area containing actin fiber. Arrows point to the actin fiber. Scale bars: protrusion image: 1 μm, inset 1: 200 nm, inset 2: 200 nm.

Biology Open • Supplementary information

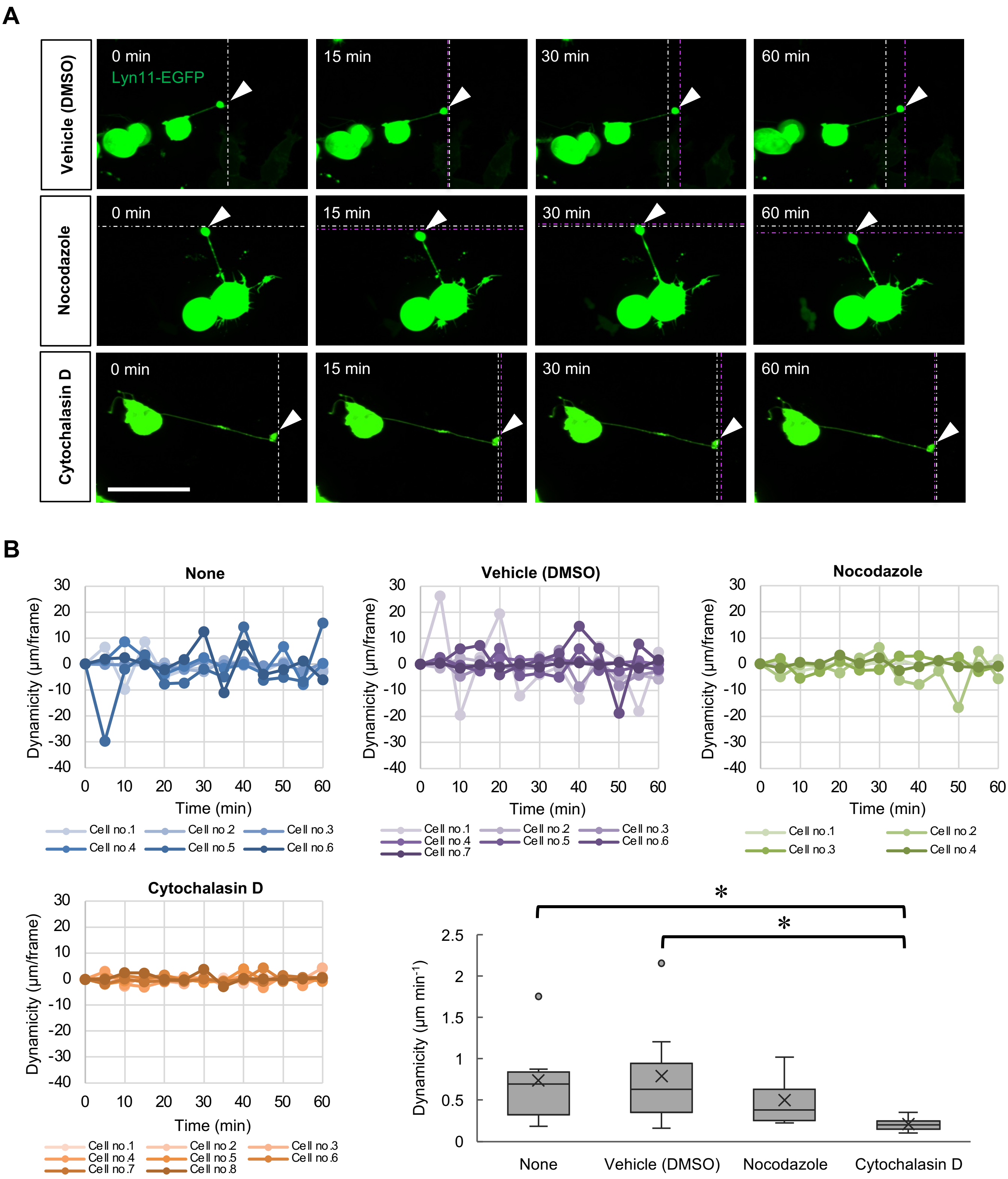

Biology Open • Supplementary information

Fig. S4. Observed protrusions were sensitive to cytochalasin D treatment

(A) Representative images from time-lapse imaging of protrusions of LAR and Lyn11-EGFP co-transfected cells. Images were acquired every 5 minutes. In vehicle (DMSO)-treated cell (upper row panels), the protrusion elongated over time, while in cytochalasin D (2 μM)-treated cell (bottom row panels), protrusion growth was inhibited. Nocodazole (2 μM)-treated cell showed some growing and shrinking dynamicity (middle row panels). The white dashed lines indicate initial location of the protrusions' tips and the magenta dashed lines indicate the location of the tips at the indicated time point. The arrowheads point to the tips of the protrusions. Green fluorescence signals are of Lyn11-EGFP co-transfected with LAR. Scale bar: 50 μm.

(B) Analytical results of the time-lapse images of protrusions from LAR and Lyn11-EGFP co-transfected cells treated with different chemicals. The growth or shrinkage rate per frame of each cell is shown in line graphs for the untreated (none), vehicle (DMSO)-treated, nocodazole-treated, and cytochalasin D-treated samples. The average dynamicity evaluated as the change in the protrusion length over time (either growth or shrinkage) among the different treatments is shown in the box plot (the horizontal line within each box denotes the median value, and the "x" mark denotes the mean value). Four independent trials were conducted. Total number of imaged cells: n = 6, 7, 4, 8 for the non-treated, vehicle-treated, nocodazole-treated, and cytochalasin D-treated groups, respectively. * $p < 0.05$, nonparametric Wilcoxon test.

Biology Open • Supplementary information

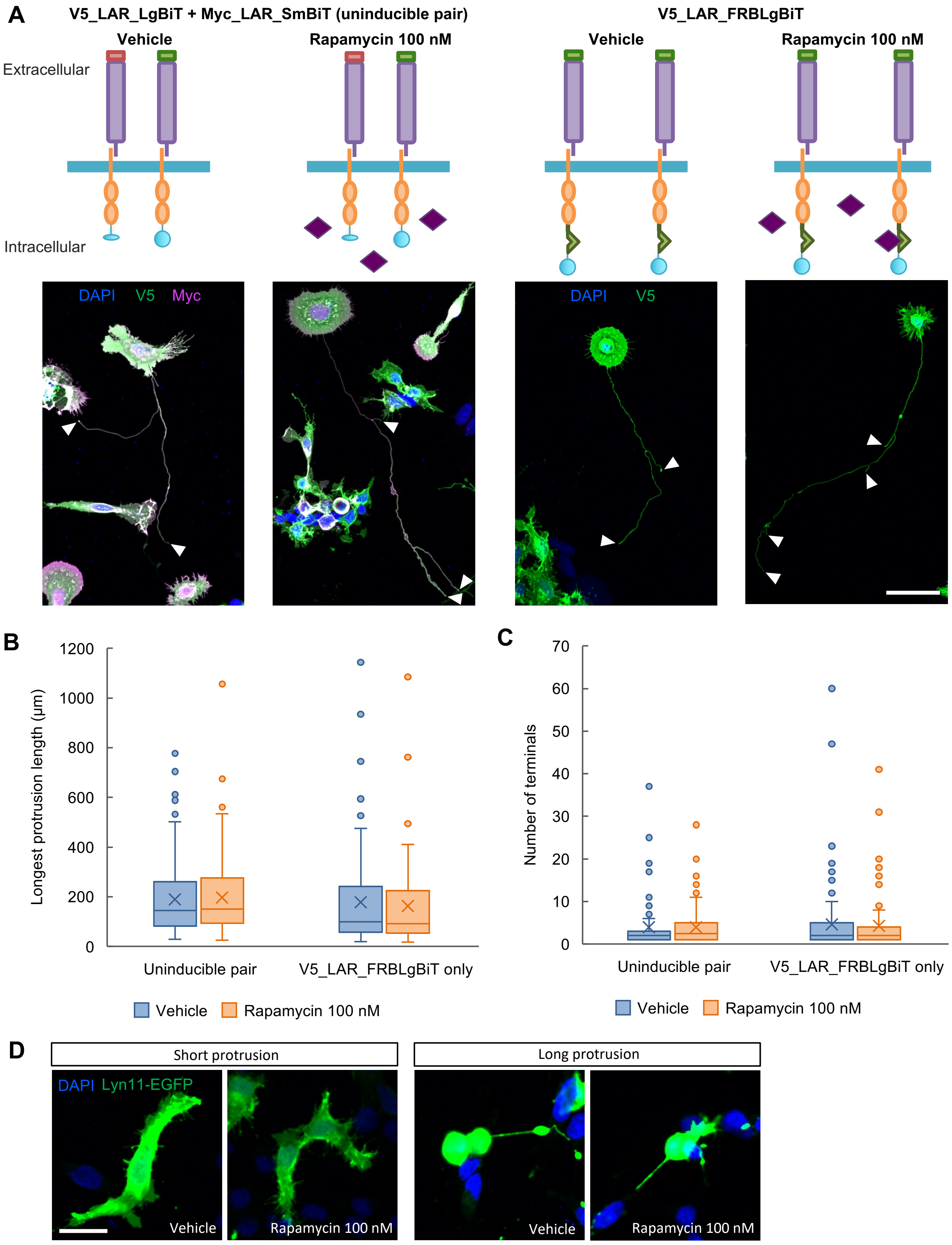
A
V5_LAR_LgBiT + Myc_LAR_SmBiT (uninducible pair)
Vehicle
Rapamycin 100 nM
V5_LAR_FRBLgBiT
Vehicle
Rapamycin 100 nM
Extracellular
Intracellular
DAPI V5 Myc
DAPI V5
B
Longest protrusion length (μm)
1200
1000
800
600
400
200
0
Uninducible pair
V5_LAR_FRBLgBiT only
Vehicle
Rapamycin 100 nM
C
Number of terminals
70
60
50
40
30
20
10
0
Uninducible pair
V5_LAR_FRBLgBiT only
Vehicle
Rapamycin 100 nM
D
Short protrusion
Long protrusion
DAPI Lyn11-EGFP
Vehicle
Rapamycin 100 nM
Vehicle
Rapamycin 100 nM

Fig. S5. No direct effect of rapamycin was observed

(A) Representative images of cells expressing the uninducible pair (V5_LAR_LgBiT+Myc_LAR_SmBiT) or V5_LAR_FRBLgBiT treated with vehicle or rapamycin (100 nM) from three independent trials. DAPI: blue, V5: green, Myc: magenta. Arrowheads: counted protrusion end tips (terminals). Scale bar: 40 μm.

(B) Analytical results of the longest protrusion length. N = 3 independent trials; total n = 82, 86, 90, and 90 cells, respectively. Nonparametric Wilcoxon test showed no significant difference. In the box plot, the horizontal line within each box denotes the median value, and the “x” mark denotes the mean value.

(C) Analytical results of the longest protrusion complexity. N = 3 independent trials; total n = 82, 86, 90, and 90 cells, respectively. Nonparametric Wilcoxon test showed no significant difference. In the box plot, the horizontal line within each box denotes the median value, and the “x” mark denotes the mean value.

(D) Representative images of short protrusions and long protrusions formed in Lyn11-EGFP-expressing cells that were treated with vehicle or rapamycin (100 nM), from two independent trials. DAPI: blue, Lyn11-EGFP: green. Scale bar: 20 μm.

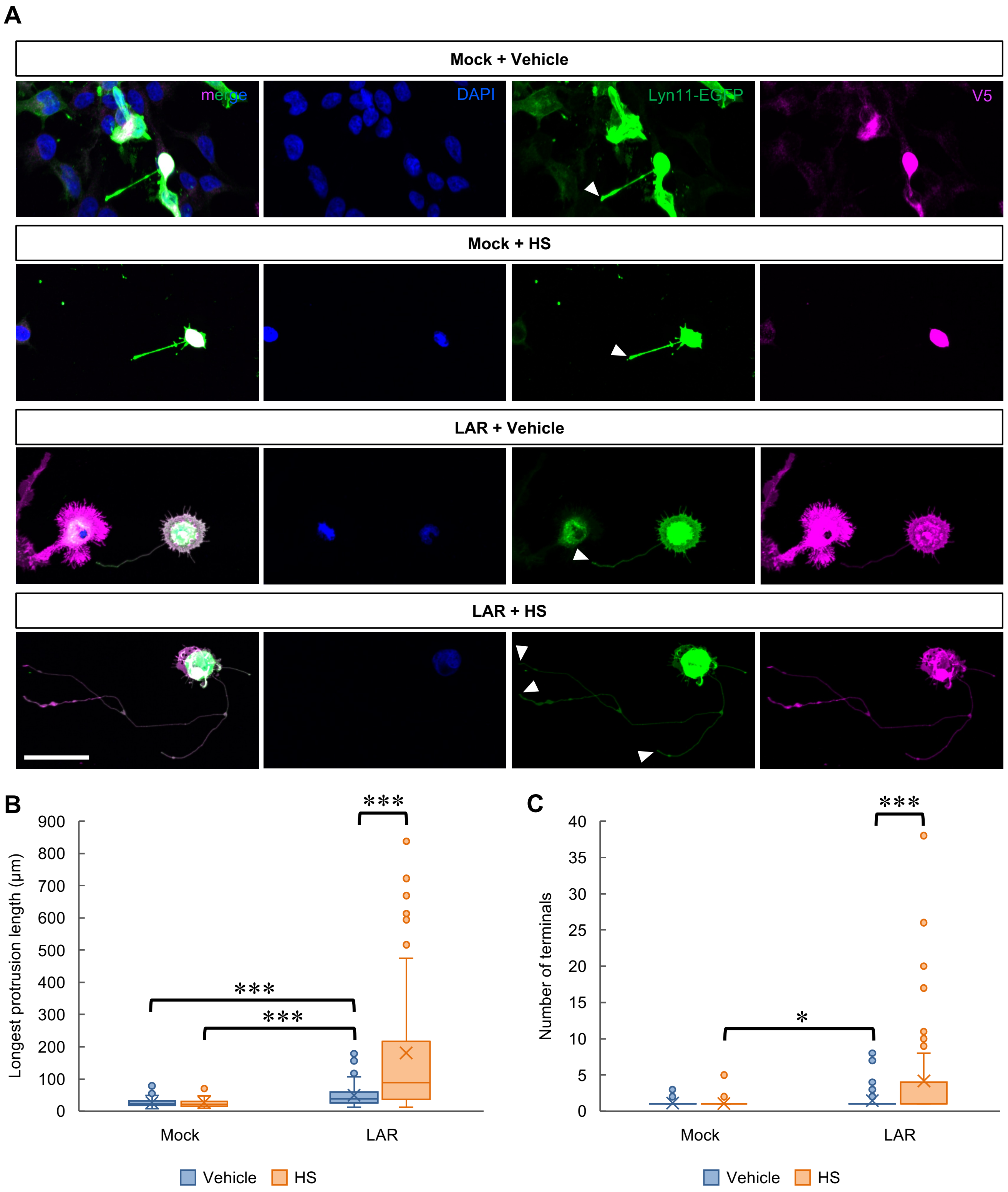

Biology Open • Supplementary information

Fig. S6. Heparan sulfate did not promote protrusion formation in mock vector-expressing cells

(A) Representative images of the cells expressing the mock or LAR vectors and treated with vehicle or heparan sulfate (HS) (those with a dominantly long protrusion) from three independent trials. DAPI: blue, Lyn11-EGFP: green, V5: magenta. Arrowheads: counted protrusion end tips (terminals). Scale bar: 40 μm.

(B) Analytical results of the longest protrusion length. HS: heparan sulfate. N = 3 independent trials; total n = 40, 55, 61, and 58 cells, respectively. *** $p < 0.001$, nonparametric Wilcoxon test. In the box plot, the horizontal line within each box denotes the median value, and the “x” mark denotes the mean value.

(C) Analytical results of the longest protrusion complexity. HS: heparan sulfate. N = 3 independent trials; total n = 40, 55, 61, and 58 cells, respectively. * $p < 0.05$ and *** $p < 0.001$, nonparametric Wilcoxon test. In the box plot, the horizontal line within each box denotes the median value, and the “x” mark denotes the mean value.

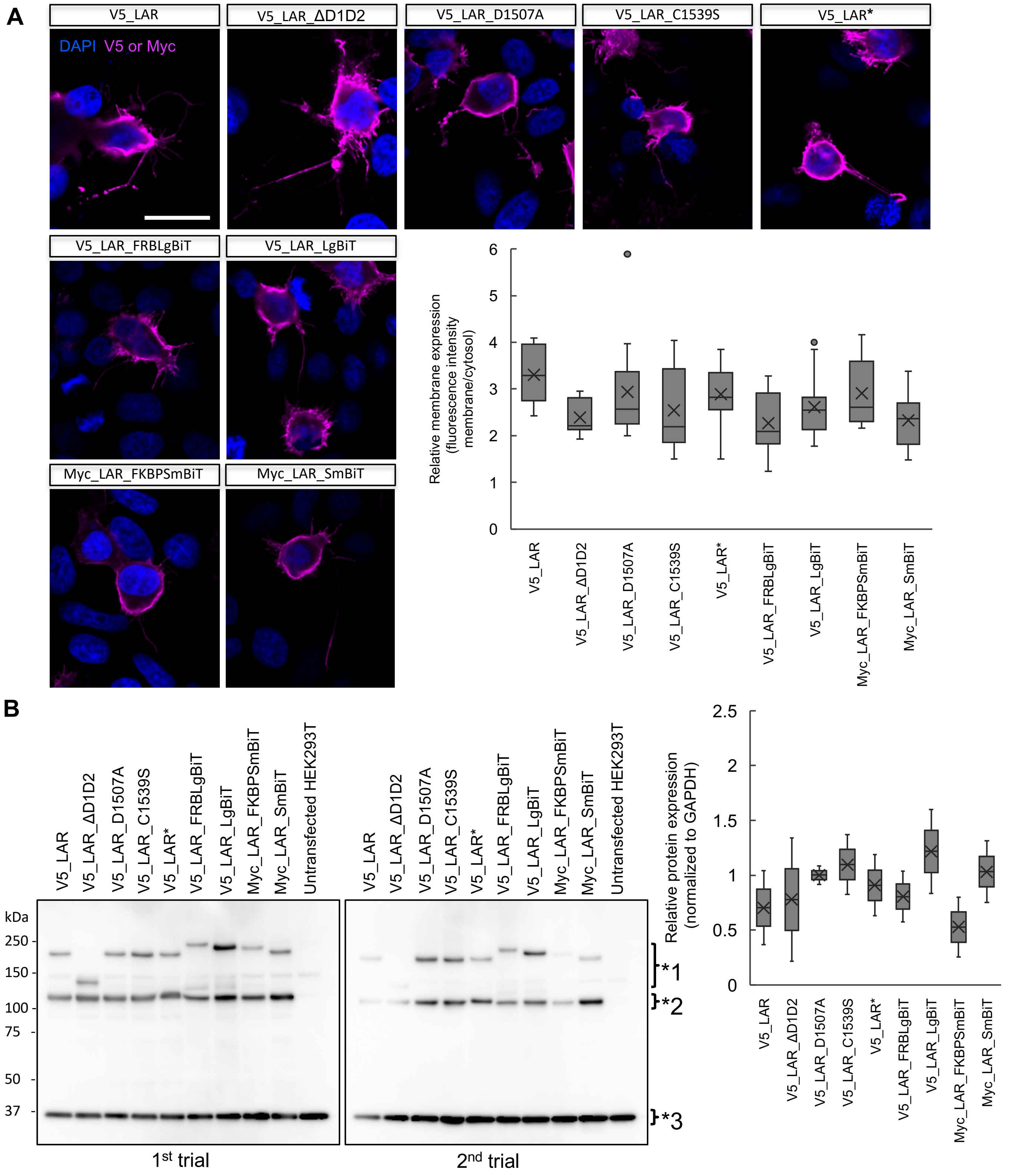

Biology Open • Supplementary information

Fig. S7. Similar membrane localization and protein expression level of the LAR constructs

(A) Analytical result of cell membrane expression of the LAR constructs, from three different culture wells for each construct, and their representative images. LAR_ΔD1D2: LAR depleted of the phosphatase-related D1 and D2 domains. LAR_D1507A: LAR with #1507 aspartic acid mutated to alanine. LAR_C1539S: LAR with #1539 cysteine mutated to serine. LAR*: LAR with K68A, K69A, K71A, K72A, R97A, and R100A mutations. DAPI: blue, V5 or Myc: magenta. Scale bar: 20 μm. n = 8, 9, 12, 9, 9, 9, 13, 9, and 8 cells, respectively. Nonparametric Steel-Dwass test showed no significant difference. In the box plot, the horizontal line within each box denotes the median value, and the “x” mark denotes the mean value.

(B) Western blot results of the LAR constructs, from two independent trials. *1 shows area where full-length LARs’ bands were detected, *2 shows area where cleaved extracellular domains of LARs were detected, and *3 shows area where endogenous GAPDH bands were detected. Nonparametric Steel-Dwass test showed no significant difference. In the box plot, the horizontal line within each box denotes the median value, and the “x” mark denotes the mean value.

Table S1. List of the primers used

Experiments/Constructs	Name	Sequence (5'-3')
LAR cloning	F-nested-PTPrF(LAR)	CCCAGCGAGAGTGACTGGTAT AAGC
	R-nested-PTPrF(LAR)	GTGTTCTGGGCTCTCTGACTGG TGT
	F-NotI-PTPrF(LAR)	ataagcggccgccATGGCTCCCGAG CCAGCCCCAG
	R-NheI-PTPrF(LAR)	ataagctagcCGTTGCATAGTGATC AAAGCTGCCGAGG
pEB_PPTsp_V5_5MCS	Forward primer	AGGGGTACCGCCACCATGTCT GCACTTCTGATCCTAGCTCTTG TTGGAGCTGCAGTTGCT
	Reverse primer	GACCTCGAGCGTAGAATCGAG ACCGAGGAGAGGGTTAGGGAT AGGCTTACCAGCAACTGC
pEB_PPTsp_Myc_5MCS	Forward primer	AGGGGTACCGCCACCATGTCT GCACTTCTGATCCTAGCTCTTG TTGGAGCTGCAGTTGCT
	Reverse primer	GACCTCGAGGAATTCAAGCTTC AGATCCTCTTCTGAGATGAGTT TTTGTTCAGCAACTGC
pEB_PPTsp_V5_LAR or pEB_PPTsp_Myc_LAR	Fwd-SalI-PTPrF(LAR)	tctagtcgacGACAGCAAACCCGTC TTTGTTAAGGTCCC
	Rev-NotI-PTPrF(LAR)	tctagcggccgcTTACGTTGCATAGT GATCAAAGCTGCCGA
pEB_PPTsp_V5_LARΔD1D2	Fwd-SalI-PTPrF(LAR)	tctagtcgacGACAGCAAACCCGTC TTTGTTAAGGTCCC
	Rev_NotI_deltaD1D2_LAR	tctagcggccgcTTAGGAATTCTCCC ATGTGAACTGCTGTCCAG
LAR-(LgBiT/SmBiT)	NanoLuc_Fwd_PTPrF(LAR)	tctagcgatcgccATGGCTCCCGAGC CAGC
	NanoLuc_Rev_PTPrF(LAR)	tctagtttaaacCGTTGCATAGTGAT CAAAGCTGCCGAGG

Biology Open • Supplementary information

Table S2. List of the LAR constructs used

Name	Related figures
pEB_PPTsp_V5_LAR	Fig. 1, 2, 6, 7, 8; Fig. S1, S2, S4, S6, S7
pEB_PPTsp_V5_LAR (K68,69,71,72A and R97,100A)	Fig. 6; Fig. S7
pEB_PPTsp_V5_LAR_ΔD1D2	Fig. 7, 8; Fig. S3, S7
pEB_PPTsp_V5_LAR_D1507A	Fig. 7, 8; Fig. S7
pEB_PPTsp_V5_LAR_C1539S	Fig. 7, 8; Fig. S7
pEB_PPTsp_V5_LAR_FRB_LgBiT	Fig. 3, 4, 5; Fig. S5, S7
pEB_PPTsp_Myc_LAR_FKBP_SmBiT	Fig. 3, 4, 5; Fig. S7
pEB_PPTsp_V5_LAR_LgBiT	Fig. 3, 5; Fig. S5, S7
pEB_PPTsp_Myc_LAR_SmBiT	Fig. 3, 5; Fig. S5, S7

Table S3. Amino acid sequence of the LARs used in this study

Name	Amino acid sequence
LAR (without signal peptide sequence)	DSKPVFVKVPEDQTGLSGGVASFVCQATGEPKPRITWMKKGKKVSSQRFEVIEFDDGAGSVLRIQPLRVQRDEAIYECTATNSLGEINTSAKLSVLEEDQLPSGFPTI<br>DMGPQLKVVEKGRTATMLCAAGGNPDPEISWFKDFLPVDPAASNGRIKQLRSGGSPIRGALQIESSEESDQGKYECVATNSAGTRYSAPANLYVRVRRVAPRFSIPP<br>SSQEVMPGGSVNLTCVAVGAPMPYVKWMMGAEELTKEDEMPVGRNVLELSNVMRSANYTCVAISSLGMIEATAQVTVKALPKPPIDLVVTETTATSVTLTWDSGNT<br>EPVSFYGIQYRAAGTDGPFQEVDGVASTRYSIGGLSPFSEYAFRVLAVNSIGRGPPSEAVRARTGEQAPSSPPRRVQARMLSASTMLVQWEPPEEPNGLVRGYRV<br>YYTPDSRRPLSAWHKHNTDAGLLTTVGSLLPGITYSLRVLAFTAVGDGPPSPTIQVKTQQGVPAQPADFQANAESDTRIQLSWLLPPQERIVKYELVYWAAEDEGQQ<br>HKVTFDPTSSYTLEDLKPDTLYHFQLAARSDLGVGVFTPTVEARTAQSMPSGPPRKVEVEPLNSTAVHVSWKLPVPNKQHGQIRGYQVTYVRLENGEPRGQPIIQDV<br>MLAEAQWRPEESEDYETTISGLTPETTYSITVAAYTTKGDGARSKPKVVTTTGAVFAKNFRVAAAMKTSVLLSWEVPDSYKSAVPFKILYNGQSVEVDGHSMRKLIAD<br>LQPNTEYSFVLMNRGSSAGGLQHLVSIRTAPDLLPQKPLPASAFIEDGRFSLSMPQVQDPSLVRWFYIVVVPIDRVGGNLLAPRWNTPEELELDELLEAIEQGEEKQR<br>RRRRQAERLKPYVAAQVDVLPDTFTLGDKKSYRGFYNRPLSPDLSYQCFVLASLKEPMDQKRYASSPYSDEIVVQVTPAQQQEEPEMLWVTGPVLAVILIILIVIAILLF<br>KRKRTHSPSSKDEQSIGLKDSLLAHSSDPVEMRRLNYQTPGSSAPSCPNISSMRDHPPIPITDLADNIERLKANDGLKFSQEYESIDPGQQFTWENSNSEVNKPKNRY<br>ANVIAYDHSRVLLTSIDGVPGSDYINANYIDGYRKQNAYIATQGPLPETMGDFWRMVWEQRTATVVMMTRLEEKSRVKCDQYWPVRGTETYGLIQVTLVDTVELATY<br>TMRTFALHKSGSSEKRELRQFQFMAWPDHGVPEYPTPILAFLRRVKACNPLDAGPMVVHCSAGVGRTGCFIVIDAMLERMKHEKTVDIYGHVTCMRSQRNYMVQTE<br>DQYVFIHEALLEAAMCGHTEVLARNLYAHIQKLGQVPPGESVTAMELEFKLLANSKAHTSRFVSANLPCNKFKNRLVNIMPYELTRVCLQPIRGVEGSDYINASFLDGY<br>RQQKAYIATQGPLAESTEDFWRMLWEHNSTIIVMLTKLREMGREKCHQYWPAERSARYQYFVVDPMAEYNMPQYILREFKVTDARDGQSRTIRQFQFTDWPEQGV<br>PKTGEGFIDFIGQVHKTKEQFGQDGPITVHCSAGVGRTGVFITLSIVLERMRYEGVVDMFQTVKTLRTQRPAMVQTEDQYQLCYRAALEYLGSFDHYAT
LAR (K68,69,71,72A and R97,100A) (without signal peptide sequence)	DSKPVFVKVPEDQTGLSGGVASFVCQATGEPKPRITWMAAGAAVSSQRFEVIEFDDGAGSVLRIQPLAVQADEAIYECTATNSLGEINTSAKLSVLEEDQLPSGFPTI<br>DMGPQLKVVEKGRTATMLCAAGGNPDPEISWFKDFLPVDPAASNGRIKQLRSGGSPIRGALQIESSEESDQGKYECVATNSAGTRYSAPANLYVRVRRVAPRFSIPP<br>SSQEVMPGGSVNLTCVAVGAPMPYVKWMMGAEELTKEDEMPVGRNVLELSNVMRSANYTCVAISSLGMIEATAQVTVKALPKPPIDLVVTETTATSVTLTWDSGNT<br>EPVSFYGIQYRAAGTDGPFQEVDGVASTRYSIGGLSPFSEYAFRVLAVNSIGRGPPSEAVRARTGEQAPSSPPRRVQARMLSASTMLVQWEPPEEPNGLVRGYRV<br>YYTPDSRRPLSAWHKHNTDAGLLTTVGSLLPGITYSLRVLAFTAVGDGPPSPTIQVKTQQGVPAQPADFQANAESDTRIQLSWLLPPQERIVKYELVYWAAEDEGQQ<br>HKVTFDPTSSYTLEDLKPDTLYHFQLAARSDLGVGVFTPTVEARTAQSMPSGPPRKVEVEPLNSTAVHVSWKLPVPNKQHGQIRGYQVTYVRLENGEPRGQPIIQDV<br>MLAEAQWRPEESEDYETTISGLTPETTYSITVAAYTTKGDGARSKPKVVTTTGAVFAKNFRVAAAMKTSVLLSWEVPDSYKSAVPFKILYNGQSVEVDGHSMRKLIAD<br>LQPNTEYSFVLMNRGSSAGGLQHLVSIRTAPDLLPQKPLPASAFIEDGRFSLSMPQVQDPSLVRWFYIVVVPIDRVGGNLLAPRWNTPEELELDELLEAIEQGEEKQR<br>RRRRQAERLKPYVAAQVDVLPDTFTLGDKKSYRGFYNRPLSPDLSYQCFVLASLKEPMDQKRYASSPYSDEIVVQVTPAQQQEEPEMLWVTGPVLAVILIILIVIAILLF<br>KRKRTHSPSSKDEQSIGLKDSLLAHSSDPVEMRRLNYQTPGSSAPSCPNISSMRDHPPIPITDLADNIERLKANDGLKFSQEYESIDPGQQFTWENSNSEVNKPKNRY<br>ANVIAYDHSRVLLTSIDGVPGSDYINANYIDGYRKQNAYIATQGPLPETMGDFWRMVWEQRTATVVMMTRLEEKSRVKCDQYWPVRGTETYGLIQVTLVDTVELATY<br>TMRTFALHKSGSSEKRELRQFQFMAWPDHGVPEYPTPILAFLRRVKACNPLDAGPMVVHCSAGVGRTGCFIVIDAMLERMKHEKTVDIYGHVTCMRSQRNYMVQTE<br>DQYVFIHEALLEAAMCGHTEVLARNLYAHIQKLGQVPPGESVTAMELEFKLLANSKAHTSRFVSANLPCNKFKNRLVNIMPYELTRVCLQPIRGVEGSDYINASFLDGY<br>RQQKAYIATQGPLAESTEDFWRMLWEHNSTIIVMLTKLREMGREKCHQYWPAERSARYQYFVVDPMAEYNMPQYILREFKVTDARDGQSRTIRQFQFTDWPEQGV<br>PKTGEGFIDFIGQVHKTKEQFGQDGPITVHCSAGVGRTGVFITLSIVLERMRYEGVVDMFQTVKTLRTQRPAMVQTEDQYQLCYRAALEYLGSFDHYAT
LAR_ΔD1D2 (without signal peptide sequence)	DSKPVFVKVPEDQTGLSGGVASFVCQATGEPKPRITWMKKGKKVSSQRFEVIEFDDGAGSVLRIQPLRVQRDEAIYECTATNSLGEINTSAKLSVLEEDQLPSGFPTI<br>DMGPQLKVVEKGRTATMLCAAGGNPDPEISWFKDFLPVDPAASNGRIKQLRSGGSPIRGALQIESSEESDQGKYECVATNSAGTRYSAPANLYVRVRRVAPRFSIPP<br>SSQEVMPGGSVNLTCVAVGAPMPYVKWMMGAEELTKEDEMPVGRNVLELSNVMRSANYTCVAISSLGMIEATAQVTVKALPKPPIDLVVTETTATSVTLTWDSGNT<br>EPVSFYGIQYRAAGTDGPFQEVDGVASTRYSIGGLSPFSEYAFRVLAVNSIGRGPPSEAVRARTGEQAPSSPPRRVQARMLSASTMLVQWEPPEEPNGLVRGYRV<br>YYTPDSRRPLSAWHKHNTDAGLLTTVGSLLPGITYSLRVLAFTAVGDGPPSPTIQVKTQQGVPAQPADFQANAESDTRIQLSWLLPPQERIVKYELVYWAAEDEGQQ<br>HKVTFDPTSSYTLEDLKPDTLYHFQLAARSDLGVGVFTPTVEARTAQSMPSGPPRKVEVEPLNSTAVHVSWKLPVPNKQHGQIRGYQVTYVRLENGEPRGQPIIQDV<br>MLAEAQWRPEESEDYETTISGLTPETTYSITVAAYTTKGDGARSKPKVVTTTGAVFAKNFRVAAAMKTSVLLSWEVPDSYKSAVPFKILYNGQSVEVDGHSMRKLIAD<br>LQPNTEYSFVLMNRGSSAGGLQHLVSIRTAPDLLPQKPLPASAFIEDGRFSLSMPQVQDPSLVRWFYIVVVPIDRVGGNLLAPRWNTPEELELDELLEAIEQGEEKQR<br>RRRRQAERLKPYVAAQVDVLPDTFTLGDKKSYRGFYNRPLSPDLSYQCFVLASLKEPMDQKRYASSPYSDEIVVQVTPAQQQEEPEMLWVTGPVLAVILIILIVIAILLF<br>KRKRTHSPSSKDEQSIGLKDSLLAHSSDPVEMRRLNYQTPGSSAPSCPNISSMRDHPPIPITDLADNIERLKANDGLKFSQEYESIDPGQQFTWENS
LAR_D1507A (without signal peptide sequence)	DSKPVFVKVPEDQTGLSGGVASFVCQATGEPKPRITWMKKGKKVSSQRFEVIEFDDGAGSVLRIQPLRVQRDEAIYECTATNSLGEINTSAKLSVLEEDQLPSGFPTI<br>DMGPQLKVVEKGRTATMLCAAGGNPDPEISWFKDFLPVDPAASNGRIKQLRSGGSPIRGALQIESSEESDQGKYECVATNSAGTRYSAPANLYVRVRRVAPRFSIPP<br>SSQEVMPGGSVNLTCVAVGAPMPYVKWMMGAEELTKEDEMPVGRNVLELSNVMRSANYTCVAISSLGMIEATAQVTVKALPKPPIDLVVTETTATSVTLTWDSGNT<br>EPVSFYGIQYRAAGTDGPFQEVDGVASTRYSIGGLSPFSEYAFRVLAVNSIGRGPPSEAVRARTGEQAPSSPPRRVQARMLSASTMLVQWEPPEEPNGLVRGYRV<br>YYTPDSRRPLSAWHKHNTDAGLLTTVGSLLPGITYSLRVLAFTAVGDGPPSPTIQVKTQQGVPAQPADFQANAESDTRIQLSWLLPPQERIVKYELVYWAAEDEGQQ<br>HKVTFDPTSSYTLEDLKPDTLYHFQLAARSDLGVGVFTPTVEARTAQSMPSGPPRKVEVEPLNSTAVHVSWKLPVPNKQHGQIRGYQVTYVRLENGEPRGQPIIQDV<br>MLAEAQWRPEESEDYETTISGLTPETTYSITVAAYTTKGDGARSKPKVVTTTGAVFAKNFRVAAAMKTSVLLSWEVPDSYKSAVPFKILYNGQSVEVDGHSMRKLIAD<br>LQPNTEYSFVLMNRGSSAGGLQHLVSIRTAPDLLPQKPLPASAFIEDGRFSLSMPQVQDPSLVRWFYIVVVPIDRVGGNLLAPRWNTPEELELDELLEAIEQGEEKQR<br>RRRRQAERLKPYVAAQVDVLPDTFTLGDKKSYRGFYNRPLSPDLSYQCFVLASLKEPMDQKRYASSPYSDEIVVQVTPAQQQEEPEMLWVTGPVLAVILIILIVIAILLF<br>KRKRTHSPSSKDEQSIGLKDSLLAHSSDPVEMRRLNYQTPGSSAPSCPNISSMRDHPPIPITDLADNIERLKANDGLKFSQEYESIDPGQQFTWENSNSEVNKPKNRY<br>ANVIAYDHSRVLLTSIDGVPGSDYINANYIDGYRKQNAYIATQGPLPETMGDFWRMVWEQRTATVVMMTRLEEKSRVKCDQYWPVRGTETYGLIQVTLVDTVELATY<br>TMRTFALHKSGSSEKRELRQFQFMAWPAHGVPEYPTPILAFLRRVKACNPLDAGPMVVHCSAGVGRTGCFIVIDAMLERMKHEKTVDIYGHVTCMRSQRNYMVQTE<br>DQYVFIHEALLEAAMCGHTEVLARNLYAHIQKLGQVPPGESVTAMELEFKLLANSKAHTSRFVSANLPCNKFKNRLVNIMPYELTRVCLQPIRGVEGSDYINASFLDGY<br>RQQKAYIATQGPLAESTEDFWRMLWEHNSTIIVMLTKLREMGREKCHQYWPAERSARYQYFVVDPMAEYNMPQYILREFKVTDARDGQSRTIRQFQFTDWPEQGV<br>PKTGEGFIDFIGQVHKTKEQFGQDGPITVHCSAGVGRTGVFITLSIVLERMRYEGVVDMFQTVKTLRTQRPAMVQTEDQYQLCYRAALEYLGSFDHYAT
LAR_C15339S (without signal peptide sequence)	DSKPVFVKVPEDQTGLSGGVASFVCQATGEPKPRITWMKKGKKVSSQRFEVIEFDDGAGSVLRIQPLRVQRDEAIYECTATNSLGEINTSAKLSVLEEDQLPSGFPTI<br>DMGPQLKVVEKGRTATMLCAAGGNPDPEISWFKDFLPVDPAASNGRIKQLRSGGSPIRGALQIESSEESDQGKYECVATNSAGTRYSAPANLYVRVRRVAPRFSIPP<br>SSQEVMPGGSVNLTCVAVGAPMPYVKWMMGAEELTKEDEMPVGRNVLELSNVMRSANYTCVAISSLGMIEATAQVTVKALPKPPIDLVVTETTATSVTLTWDSGNT<br>EPVSFYGIQYRAAGTDGPFQEVDGVASTRYSIGGLSPFSEYAFRVLAVNSIGRGPPSEAVRARTGEQAPSSPPRRVQARMLSASTMLVQWEPPEEPNGLVRGYRV<br>YYTPDSRRPLSAWHKHNTDAGLLTTVGSLLPGITYSLRVLAFTAVGDGPPSPTIQVKTQQGVPAQPADFQANAESDTRIQLSWLLPPQERIVKYELVYWAAEDEGQQ<br>HKVTFDPTSSYTLEDLKPDTLYHFQLAARSDLGVGVFTPTVEARTAQSMPSGPPRKVEVEPLNSTAVHVSWKLPVPNKQHGQIRGYQVTYVRLENGEPRGQPIIQDV<br>MLAEAQWRPEESEDYETTISGLTPETTYSITVAAYTTKGDGARSKPKVVTTTGAVFAKNFRVAAAMKTSVLLSWEVPDSYKSAVPFKILYNGQSVEVDGHSMRKLIAD<br>LQPNTEYSFVLMNRGSSAGGLQHLVSIRTAPDLLPQKPLPASAFIEDGRFSLSMPQVQDPSLVRWFYIVVVPIDRVGGNLLAPRWNTPEELELDELLEAIEQGEEKQR<br>RRRRQAERLKPYVAAQVDVLPDTFTLGDKKSYRGFYNRPLSPDLSYQCFVLASLKEPMDQKRYASSPYSDEIVVQVTPAQQQEEPEMLWVTGPVLAVILIILIVIAILLF<br>KRKRTHSPSSKDEQSIGLKDSLLAHSSDPVEMRRLNYQTPGSSAPSCPNISSMRDHPPIPITDLADNIERLKANDGLKFSQEYESIDPGQQFTWENSNSEVNKPKNRY<br>ANVIAYDHSRVLLTSIDGVPGSDYINANYIDGYRKQNAYIATQGPLPETMGDFWRMVWEQRTATVVMMTRLEEKSRVKCDQYWPVRGTETYGLIQVTLVDTVELATY<br>TMRTFALHKSGSSEKRELRQFQFMAWPDHGVPEYPTPILAFLRRVKACNPLDAGPMVVHSSAGVGRTGCFIVIDAMLERMKHEKTVDIYGHVTCMRSQRNYMVQTE<br>DQYVFIHEALLEAAMCGHTEVLARNLYAHIQKLGQVPPGESVTAMELEFKLLANSKAHTSRFVSANLPCNKFKNRLVNIMPYELTRVCLQPIRGVEGSDYINASFLDGY<br>RQQKAYIATQGPLAESTEDFWRMLWEHNSTIIVMLTKLREMGREKCHQYWPAERSARYQYFVVDPMAEYNMPQYILREFKVTDARDGQSRTIRQFQFTDWPEQGV<br>PKTGEGFIDFIGQVHKTKEQFGQDGPITVHCSAGVGRTGVFITLSIVLERMRYEGVVDMFQTVKTLRTQRPAMVQTEDQYQLCYRAALEYLGSFDHYAT

Biology Open • Supplementary information

Table S4. List of the primary antibodies used

Name	Production company	Catalog number	Dilution ratio
DAPI solution	Dojindo (Kumamoto, Japan)	D523	1:1000
chicken anti-GFP	Abcam (Cambridge, UK)	ab13970	1:1000
mouse anti-V5	Invitrogen/Thermo Scientific (Massachusetts, USA)	R960-25	1:2000
rabbit anti-V5	MBL (Tokyo, Japan)	PM003	1:2000
rabbit anti-c-Myc (A14)	Santa Cruz Biotechnology (California, USA)	sc-789	1:200
rabbit anti-Myc	MBL (Tokyo, Japan)	562	1:200
mouse anti-alpha tubulin	Sigma-Aldrich (Munich, Germany)	T9026	1:500
Phalloidin-iFluor 555	Abcam (Cambridge, UK)	ab176756	1:1000
anti-GAPDH-HRP	Wako Pure Chemical Industries, Ltd. (Osaka, Japan)	015-25473	1:2000
mouse monoclonal anti-LAR(7)	Santa Cruz Biotechnology (California, USA)	sc-135969	1:200

Table S5. List of the secondary antibodies used

Name	Production company	Catalog number	Dilution ratio
Alexa Fluor® 488 goat anti-chicken IgG (H+L)	Invitrogen/Thermo Scientific (Massachusetts, USA)	A11039	1:2000
Alexa Fluor® 488 goat anti-mouse IgG (H+L)	Invitrogen/Thermo Scientific (Massachusetts, USA)	A11001	1:2000
Alexa Fluor® 568 goat anti-mouse IgG (H+L)	Invitrogen/Thermo Scientific (Massachusetts, USA)	A11004	1:2000
Alexa Fluor® 568 goat anti-rabbit IgG (H+L)	Invitrogen/Thermo Scientific (Massachusetts, USA)	A11011	1:2000
Cy5® goat anti-rabbit IgG (H+L)	Invitrogen/Thermo Scientific (Massachusetts, USA)	A10523	1:2000
Anti-mouse IgG, HRP-linked antibody	Cell Signaling Technology (Massachusetts, USA)	7076S	1:2000

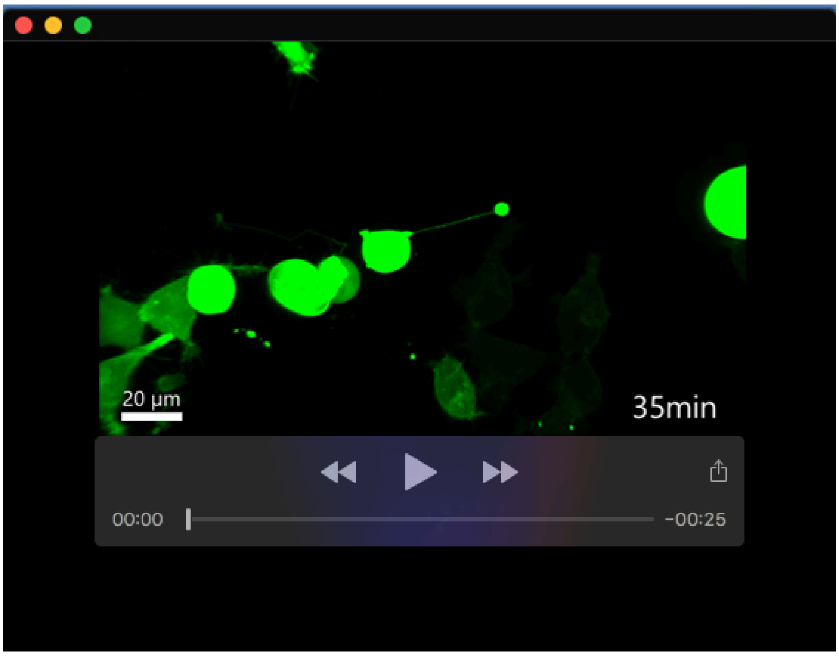

Movie 1. LAR and Lyn11-EGFP co-transfected cell treated with vehicle (DMSO)

In the vehicle (DMSO)-treated cell, the observed longest protrusion elongated over time. Images were acquired every 5 minutes. Green fluorescence signals are of Lyn11-EGFP co-transfected with LAR. Scale bar: 20 μm.

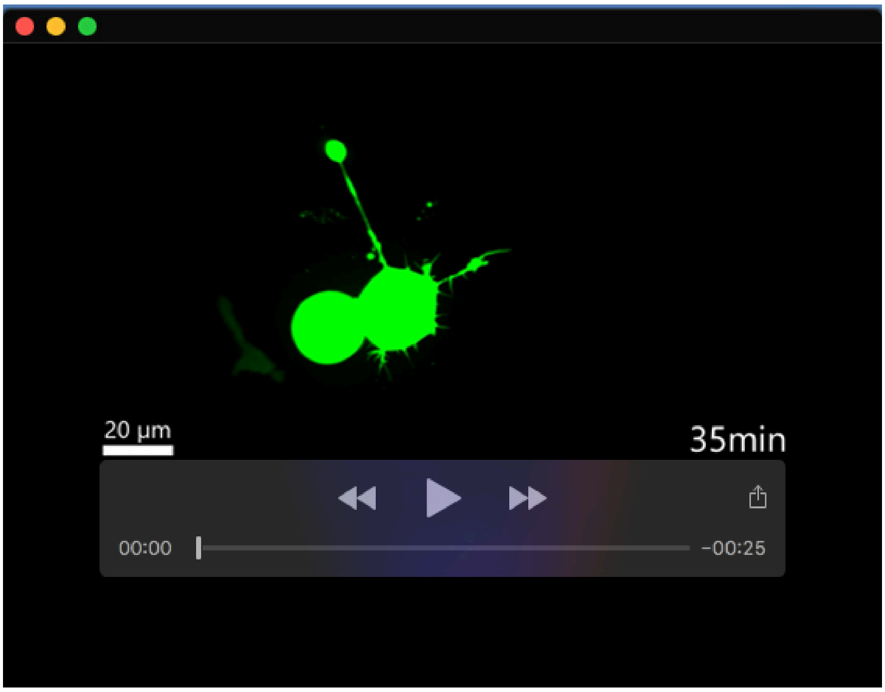

Movie 2. LAR and Lyn11-EGFP co-transfected cell treated with nocodazole

In nocodazole (2 μM)-treated cell, protrusion showed growing and shrinking dynamicity. Images were acquired every 5 minutes. Green fluorescence signals are of Lyn11-EGFP co-transfected with LAR. Scale bar: 20 μm.

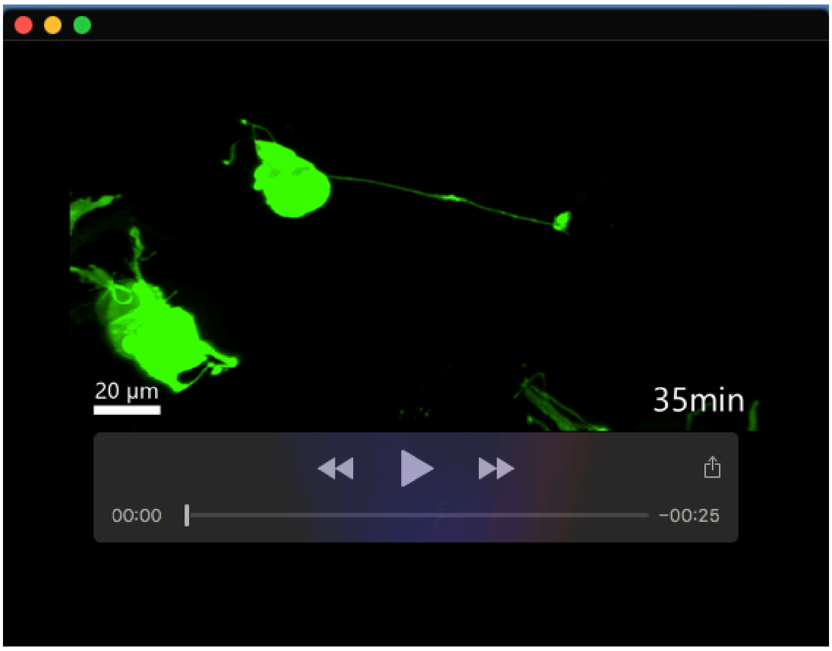

Movie 3. LAR and Lyn11-EGFP co-transfected cell treated with cytochalasin D

In cytochalasin D (2 µM)-treated cell, protrusion growth was inhibited. Images were acquired every 5 minutes. Green fluorescence signals are of Lyn11-EGFP co-transfected with LAR. Scale bar: 20 µm.

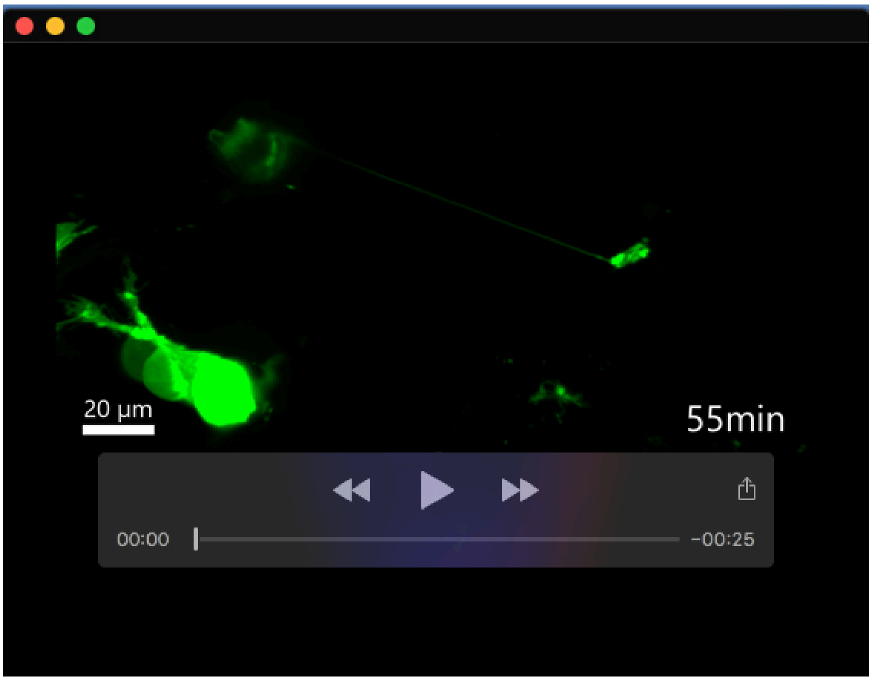

Movie 4. LAR and Lyn11-EGFP co-transfected cell treated with cytochalasin D after washout

The inhibitory effects of cytochalasin D were attenuated after washout, and the longest protrusion of the same cell shown in Movie 3 was able to elongate again. Images were acquired every 5 minutes. Green fluorescence signals are of Lyn11-EGFP co-transfected with LAR. Scale bar: 20 µm.

Biology Open • Supplementary information
